# PRC1-independent binding and activity of RYBP on the KSHV genome during *de novo* infection

**DOI:** 10.1371/journal.ppat.1010801

**Published:** 2022-08-26

**Authors:** See-Chi Lee, Zsolt Toth

**Affiliations:** 1 Department of Oral Biology, University of Florida College of Dentistry, Gainesville, Florida, United States of America; 2 UF Genetics Institute, Gainesville, Florida, United States of America; 3 UF Health Cancer Center, Gainesville, Florida, United States of America; University of Southern California, UNITED STATES

## Abstract

Kaposi’s sarcoma-associated herpesvirus (KSHV) is an oncogenic virus that causes lifelong infection in humans by establishing latency after primary infection. Latent infection is a prerequisite for both persistent infection and the development of KSHV-associated cancers. While viral lytic genes are transiently expressed after primary infection, their expression is significantly restricted and concomitant with the binding of host epigenetic repressors Polycomb Repressive Complex 1 and 2 (PRC1 and PRC2) to lytic genes. PRC1 and PRC2 mediate the repressive histone marks H2AK119ub and H3K27me3, respectively, and maintain heterochromatin structure on KSHV lytic genes to inhibit their expression. In contrast to PRC2, little is known about the recruitment and role of PRC1 factors on the KSHV genome following *de novo* infection. Thus, the goal of this study was to examine the function of PRC1 factors in the establishment of KSHV latency. To address this question, we performed an shRNA screen targeting 7 different components of the canonical and non-canonical PRC1 complexes during primary KSHV infection. We found that RYBP, a main subunit of the non-canonical PRC1 complexes, is a potent repressor of KSHV lytic genes that can bind to the viral genome and inhibit lytic genes as early as 4 hours post infection. Surprisingly, our ChIP analyses showed that RYBP binds to lytic viral gene promoters in a PRC1-independent manner, does not affect PRC1 activity on the KSHV genome, and can reduce the level of histone marks associated with transcription elongation. Our data also suggest that RYBP can repress the viral lytic cycle after primary infection by inhibiting the transcription elongation of the lytic cycle inducer KSHV gene RTA. Based on our results we propose that RYBP uses a PRC1-independent mechanism to block KSHV RTA expression thereby promoting the establishment of KSHV latency following *de novo* infection.

## Author summary

It has been shown that cellular epigenetic factors play an important role in regulating the chromatin structure of KSHV DNA by controlling the expression of viral genes following primary infection. The repression of lytic genes during latency is modulated by various inhibitory epigenetic mechanisms, including PRC1- and PRC2-mediated chromatin modifications. In this study, we identified RYBP, a major component of many PRC1 complexes, as a novel repressor of KSHV lytic genes during primary KSHV infection. Previous studies have shown that RYBP regulates the binding and enzymatic activity of PRC1 complexes, yet surprisingly, our data indicates that both the binding and repressive function of RYBP on KSHV lytic genes are PRC1-independent. We found that RYBP can inhibit the transcription elongation of the lytic cycle inducer viral gene RTA during *de novo* infection, thereby promoting the establishment of KSHV latency. Altogether, our study revealed a novel, non-canonical role of RYBP in the regulation of KSHV gene silencing during *de novo* viral infection.

## Introduction

Kaposi’s sarcoma-associated herpesvirus (KSHV) is a 165-kb double stranded DNA virus that is the etiologic agent of KSHV-associated inflammatory cytokine syndrome (KICS) and several cancers such as Kaposi’s sarcoma, Primary Effusion Lymphoma, and Multicentric Castleman’s Disease [1,2]. Like other herpesviruses, the life cycle of KSHV consists of two distinct phases: latent and lytic. Establishment of viral latency following primary infection is particularly important for KSHV pathogenesis and is a prerequisite for both lifelong infection and the development of KSHV-associated cancers. During latency, the transcription of most viral genes is repressed and there is no virus production, thereby limiting immune detection of KSHV and promoting persistent infection of the host. More importantly, the immune evasion of latently infected cells also hampers the development of KSHV specific antiviral treatments [3]. Therefore, a better understanding of how latent infection is regulated by viral and host factors is essential to identify targets for antiviral therapies against latent KSHV infection.

Following *de novo* infection, KSHV’s linear genome is circularized and rapidly becomes a chromatinized episome in the nucleus of infected cells [4,5]. Previous studies revealed that in the first 24 hours of infection (prior to the establishment of latency), the viral genome acquires a transcriptionally active chromatin with activating histone marks (e.g. H3K4me3, histone acetylation) that are associated with transient lytic viral gene expression [4,6,7]. However, after 24 hours post infection (hpi), a transcriptionally repressive chromatin forms on the KSHV genome and is accompanied by the downregulation of viral lytic gene expression [4,6,7]. Several epigenetic mechanisms such as DNA methylation, chromatin remodeling, and the regulation of histone modifications on the viral chromatin have been implicated in the inhibition of lytic viral gene expression and subsequent establishment and maintenance of viral latency following primary infection [4,8–14]. While lytic genes have many transcriptional repressors, Polycomb group proteins in particular have been shown to bind to KSHV lytic genes genome-wide and are crucial for the inhibition of lytic genes [4,10,15,16]. Polycomb proteins are evolutionarily conserved epigenetic repressors that control gene transcription through their chromatin modifying activity [17]. Originally identified in *Drosophila* as regulators of HOX gene silencing, they are now known to be essential for long-term gene repression for embryonic development, stem cell differentiation, and tissue homeostasis in both vertebrates and invertebrates [18,19]. Polycomb proteins form two distinct complexes, Polycomb Repressive Complex 1 and 2 (PRC1 and PRC2), which have different enzymatic activities. While PRC1 possesses E3 ubiquitin ligase activity and catalyzes the mono-ubiquitination of lysine residue 119 on histone H2A (H2AK119ub), PRC2 has a methyltransferase subunit that mediates the tri-methylation of histone H3 at lysine 27 (H3K27me3) [20,21]. Several studies have shown that HIV and herpesviruses other than KSHV can also utilize these PRCs to maintain heterochromatin on their viral genomes, thereby silencing viral genes to promote latent infection of their hosts [22–25].

**Fig 1:**
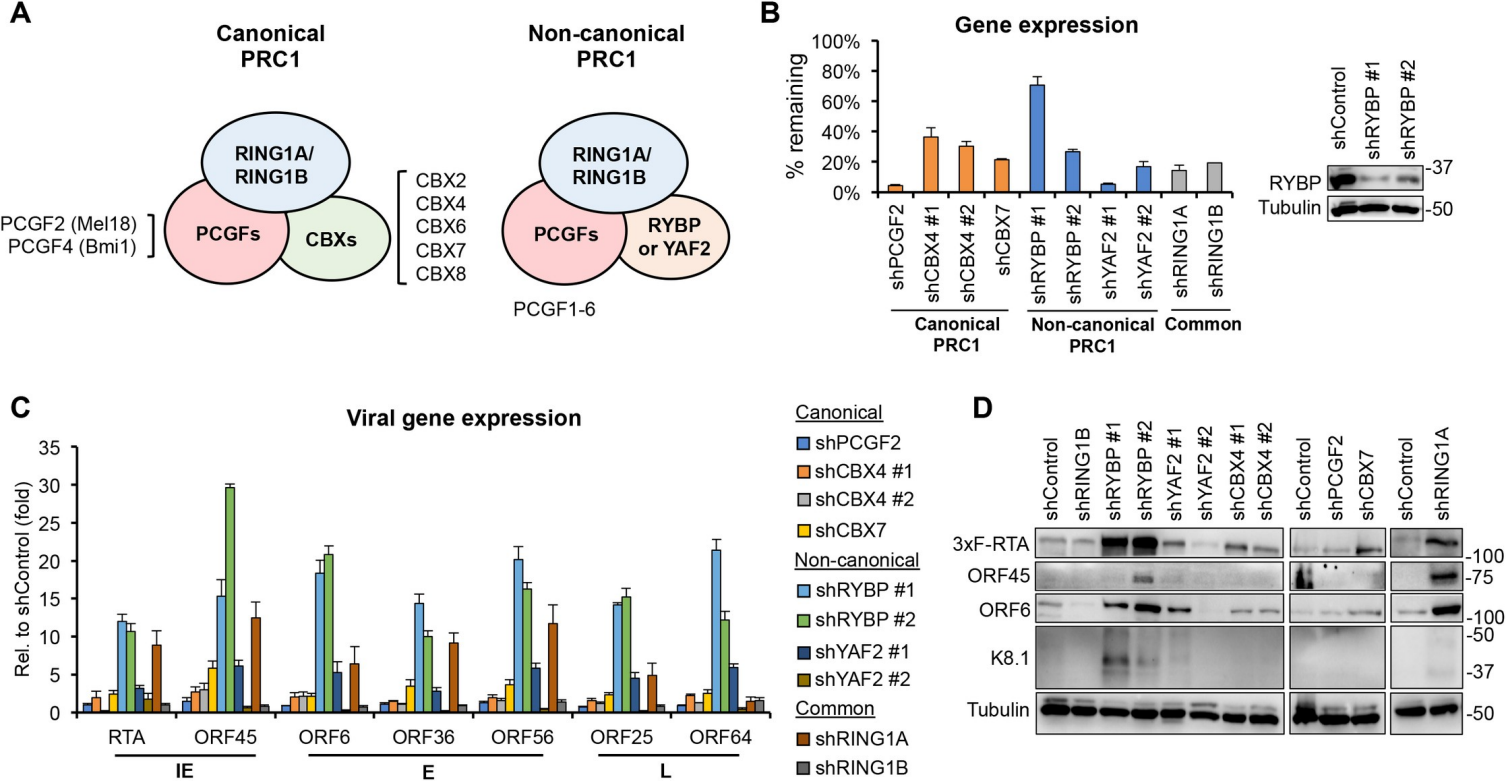
Analysis of viral gene expression changes upon shRNA knockdown of PRC1 factors during primary KSHV infection. **(A)** Components of the canonical and non-canonical Polycomb Repressive Complex 1. **(B-D)** SLK cells were transduced with lentiviruses expressing shRNAs that were specific for different subunits of PRC1. Three days post-transduction, the cells were infected with KSHV for 72 hours. **(B)** RT-qPCR and immunoblot analyses of the expression of PRC1 factors after shRNA knockdown. The percentage of remaining transcript expression is shown relative to the shControl. **(C)** RT-qPCR analysis of the expression of viral genes including immediate early (IE), early (E), and late (L) genes. **(D)** Immunoblot analysis of viral protein expression. Tubulin was used as a loading control.

Compared to PRC2, PRC1 components can form a diverse array of complexes. The core of PRC1 complexes consists of RING1A or RING1B, which are the E3 ubiquitin ligases for H2AK119ub, and one of the six polycomb group ring finger proteins (PCGF1-6), which stabilizes the enzymatic activity of PRC1 (Fig 1A) [26,27]. Depending on the composition of co-factors that bind to the core of PRC1, PRC1 complexes can further be divided into canonical and non-canonical PRC1 (cPRC1 and ncPRC1). cPRC1 includes either PCGF2 or PCGF4 and a Chromobox protein (CBX2, 4, 6–8), and can be recruited to its target promoters through PRC2-mediated H3K27me3 binding. In contrast, ncPRC1 binds to promoters in a H3K27me3-independent manner by interacting with DNA-binding transcription factors (e.g., KDM2B, YY1, MGA) [28–30]. ncPRC1 contains one of the six PCGFs and either RING1 and YY1 Binding Protein (RYBP) or YY1 Associated Factor 2 (YAF2). Importantly, the binding of RYBP/YAF2 and CBXs to RING1A/B proteins is mutually exclusive, which allows cPRC1 and ncPRC1 to form [26,31–33].

Previous studies have shown that the core PRC1 binds to the KSHV genome during latent infection and that is has a repressive effect on lytic genes. However, the role of PRC1 factors in the establishment of KSHV latency is still largely unknown. As a result, we investigated what canonical, non-canonical, and core PRC1 factors are critical for downregulating the expression of lytic genes during *de novo* KSHV infection, which is an essential step in the establishment of viral latency. Here, we identified RYBP as a novel repressor of the KSHV lytic cycle during primary infection. Additionally, we found that the binding of RYBP and its effect on viral chromatin during *de novo* infection is PRC1-independent. Our results show that RYBP can promote the establishment of viral latency by inhibiting RTA expression through repression of viral transcription elongation of the RTA gene.

## Materials and methods

### Cell lines and KSHV infection

HEK293T (ATCC), EA.hy926 (ATCC), HeLa (NIH AIDS Reagent Program) and SLK (NIH AIDS Reagent Program) were maintained in DMEM supplemented with 10% fetal bovine serum (FBS) and penicillin-streptomycin (P/S). BCBL1 (NIH AIDS Reagent Program) was cultured in RPMI containing 10% FBS and P/S. KSHV (BAC16 clone) was received from Jae U. Jung (University of Southern California). We made the KSHV clone BAC16-3xFLAG-RTA by epitope tagging the RTA gene with 3xFLAG at its 5’ end. RTA-KO KSHV was made by introducing a STOP codon in the 5’ end of RTA open reading frame, which abolishes RTA protein expression [34]. KSHV BAC16 production and viral infection were performed as described previously [13].

### shRNA knockdown and lentiviruses

The shRNAs were expressed from the lentiviral pLKO.1 vector. The target sequences of shRNAs are listed in S1 Table. In the NZFm RYBP mutant, threonine and phenylalanine at position 13 and 14 were changed to alanine. The dRBD RYBP mutant was generated by deleting residues 160–174. The RYBP truncation mutants including N158 (expressing residues 1 to 158), N79 (residues 1 to 79), and amino acid (aa) 80–158 were made by PCR and In-Fusion cloning. The RYBP phosphorylation mutants were generated by mutating designated serine residues to alanine. All RYBP mutants were expressed as N-terminal 3xFLAG-tagged proteins from the pCDHCMV-MCS-EF1 puro lentiviral vector. Lentivirus production and transduction were performed as described previously [13]. Two days after lentivirus transduction, the cells were split, and the same number of lentivirus-transduced cells was infected with KSHV.

### Antibodies and inhibitors

The antibodies used for ChIPs and immunoblot analyses are summarized in S2 Table. PTC209 and AZD4573 were purchased from Selleckchem.com (Houston, TX, United States).

### siRNA transfection

The siRNA targeting RYBP (sc-106751) was purchased from Santa Cruz Biotechnology. Lipofectamine RNAiMAX (Invitrogen) was used for siRNA transfections, which were performed according to the manufacturer’s instructions.

### Total RNA, DNA isolation and their qPCR analysis

The RNA and DNA extraction and qPCR analyses were performed as described in our previous publication [13]. RT-qPCR analysis was normalized to 18S RNA expression. The viral DNA was measured by using KSHV ORF11 specific primers. Viral DNA was normalized to cellular DNA level, which was measured by using HS1 specific primers. The primers for KSHV and the host genes are listed in 5’ to 3’ orientation in S3 Table. The RT-qPCR and qPCR results were calculated as an average of three independent experiments. To test statistical significance, we used a two-tailed Student’s t-test where *p*<0.05 was considered significant.

### Chromatin Immunoprecipitation (ChIP) assay

The ChIP assay was performed as previously described [13]. The ChIP graphs show the average of three independent ChIP experiments, which were calculated as the percentage of the immunoprecipitated DNA relative to input DNA. The ChIP antibodies are listed in S2 Table while the ChIP-qPCR primer sequences are shown in 5’ to 3’ orientation in S3 Table.

### Formaldehyde-assisted isolation of regulatory elements (FAIRE) assay

The FAIRE analysis was performed as previously described [4]. DNA was extracted from both formaldehyde-crosslinked and de-crosslinked chromatins and analyzed by qPCR using KSHV gene specific primers. DNA purified from the formaldehyde-crosslinked chromatin represents chromatin-free DNA, while DNA extracted from de-crosslinked chromatin represent total DNA. The ratio of chromatin-free DNA and total DNA can show the level of chromatinization of viral genomic regions.

### Co-immunoprecipitation assay

Transfected HEK293T cells were harvested at 48 h post-transfection. Cells were washed once with cold PBS, lysed in NP-40 lysis buffer (50 mM Tris, pH 7.5, 120 mM NaCl, 0.5% NP-40) containing protease inhibitor cocktail (Roche), and passed through a 23-gauge needle 10 times. The cell lysates were then incubated on ice for 30 min. After centrifugation, the cell lysates were subjected to preclearing using Protein A Sepharose overnight at 4°C. The next day, the cell lysates were incubated with antibodies for 3 hours at 4°C. Afterwards, Protein A/G Sepharose was added to the lysates and were further incubated for 2 hours at 4°C. Immunoprecipitates (IP) were washed three times with the lysis buffer, and the IPs were resuspended in 50 μl of 2x Laemmli buffer. Both input and IP samples were then analyzed by immunoblotting.

### Immunofluorescence assay

HeLa cells were transfected with 3xFLAG-RYBP and its mutants for two days. The cells were then washed with PBS and prepared for IF analysis, which was performed as described previously [12].

## Results

### Identification of RYBP as a major repressor of lytic genes during *de novo* KSHV infection

Previous studies have shown the genome-wide binding of RING1B and the deposition of H2AK119ub on the promoters of lytic viral genes by 72 hpi during *de novo* KSHV infection [4,16]. However, the role of PRC1 factors in the repression of lytic genes following *de novo* infection has not been investigated, and thus their function during the establishment of KSHV latency is still largely unknown. To test the role of PRC1 during *de novo* KSHV infection, we first assessed how inhibiting PRC1 activity in KSHV-infected SLK cells affects viral gene expression (S1 Fig). We used PTC209, a potent and selective PCGF4 (BMI1) inhibitor that blocks PRC1’s enzymatic activity [35]. We treated SLK cells with different concentration of PTC209 or DMSO (negative control) for 24 hours and then infected the cells with KSHV (BAC16-3xFLAG-RTA) for 72 hours. We found that PTC209 increased KSHV lytic gene transcription by 3- to 12-fold (S1A Fig), and induced lytic viral protein production (S1B Fig) indicating that PRC1 is involved in the repression of lytic genes following KSHV infection.

Next, we aimed to determine what PRC1 factors are critical for the inhibition of lytic gene expression during the establishment of KSHV latency. To do so, we performed an shRNA screen in which we targeted 7 different PRC1 factors including canonical, non-canonical, and common PRC1 subunits during KSHV (BAC16-3xFLAG-RTA) infection (Fig 1). The efficiency of shRNA knockdowns was measured by RT-qPCR and immunoblotting (Fig 1B). We note that although shRYBP #1 did not reduce RYBP mRNA expression as efficiently as shRYBP #2 but both shRNAs could reduce RYBP expression at the protein level suggesting that shRYBP #1 may repress only RYBP translation. While many of the shRNAs induced viral lytic gene expression and protein production at various levels at 72 hpi, RYBP shRNAs induced viral gene expression the strongest, by 10- to 30-fold (Fig 1C) as well as IE (RTA), E (ORF45 and ORF6), and L (K8.1) KSHV protein expression (Fig 1D). We note that although both YAF2 shRNAs could greatly reduce YAF2 expression, only shYAF2 #1 increased lytic viral gene expression substantially. However, it is possible that shYAF2 #2 knockdown efficiency was not sufficient or that it had off-target effects that interfered with KSHV lytic gene expression. To ensure that RYBP-mediated repression of lytic viral genes is not cell type specific, we also tested the effect of RYBP on viral gene expression during *de novo* KSHV infection in 293T epithelial cells and EA.hy926 endothelial cells as well as in latently infected KSHV^+^ B cell lymphoma cells (BCBL1) (S2 Fig). We found that shRYBP also induced lytic viral gene expression in these cell lines. We note that shRYBP #2 was used in these and the subsequent experiments because we found that it could inhibit RYBP expression the most consistently and robustly. Taken together, these experiments revealed that among the PRC1 factors, RYBP functions as a major repressor of viral lytic genes during primary KSHV infection and that it may also play a role in the inhibition of lytic genes during latency.

### RYBP-mediated repression of KSHV lytic cycle occurs as early as 4 hpi following viral infection and depends on the expression of RTA

**Fig 2:**
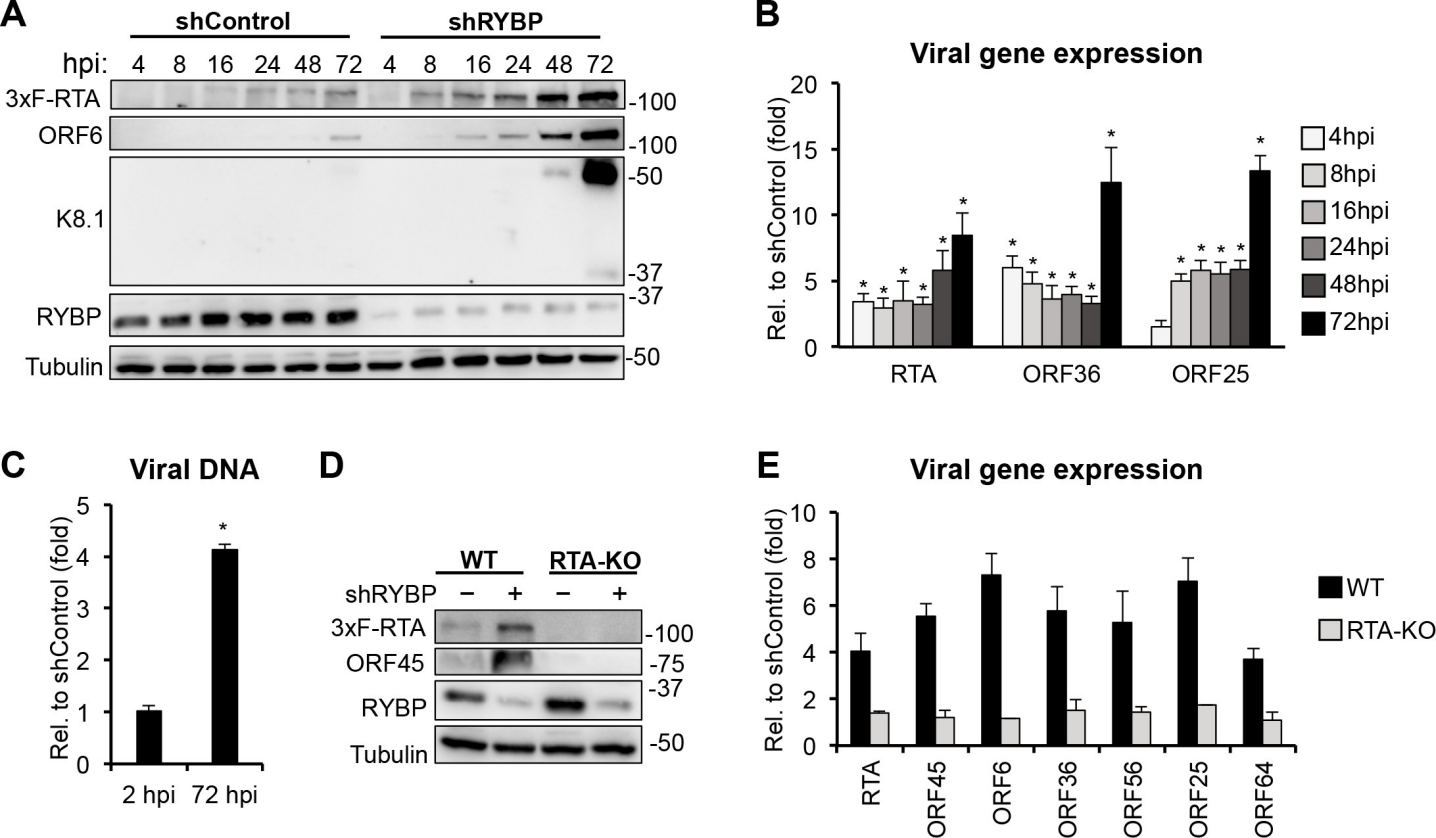
RYBP inhibits KSHV lytic genes during *de novo* infection, which are dependent on RTA expression. **(A-B**) shControl- and shRYBP-treated SLK cells were infected with BAC16-3xFLAG-RTA KSHV for 4, 8, 16, 24, 48, and 72 hours. **(A)** Immunoblot analysis of RYBP and viral protein expression. **(B)** RT-qPCR analysis of viral gene expression. The relative fold change represents the induction of viral gene expression in shRYBP-treated cells relative to shControl-treated cells at different time points of KSHV infection. **(C)** Viral DNA level at 72 hpi was measured by qPCR, normalized to the host DNA level, and then calculated relative to 2 hpi in shControl- and shRYBP-treated SLK cells. **(D-E)** shControl- and shRYBP-treated SLK cells were infected with wild-type (WT) and RTA knockout (RTA-KO) KSHV for 72 hours. **(D)** Immunoblots analysis of RYBP and viral protein expression. **(E)** Measuring viral gene expression by RT-qPCR. The relative fold change represents the induction of viral gene expression in shRYBP-treated cells relative to shControl-treated cells.

To determine the kinetics of RYBP-mediated viral gene repression during *de novo* KSHV infection, shControl- and shRYBP-treated SLK cells were infected with KSHV (BAC16-3xFLAG-RTA) and viral protein and gene expression were analyzed at 4, 8, 16, 24, 48, and 72 hpi (Fig 2A and 2B). We observed increased lytic gene expression (2- to 6-fold) and viral protein production in shRYBP cells compared to shControl cell as early as 4 hpi, which were the most pronounced at 72 hpi (Fig 2A and 2B). We also found that while the viral DNA level was comparable between KSHV-infected shControl and shRYBP-treated SLK cells at 2 hpi, it increased 4-fold in shRYBP-treated cells as compared to shControl-treated cells at 72 hpi (Fig 2C). Taken together, these data indicate that RYBP can restrict the KSHV lytic cycle as early as 4 hpi, and that it is required for blocking KSHV lytic replication following *de novo* infection.

It is well established that RTA is an essential viral transcription factor of KSHV that initiates and drives the lytic gene expression cascade, although some lytic genes can also be induced independently of RTA. Therefore, we tested whether shRYBP-mediated induction of lytic genes requires RTA expression. We infected shControl- and shRYBP-treated SLK cells with WT KSHV (BAC16-3xFLAG-RTA) or RTA-KO KSHV (BAC16-3xFLAG-RTAstop) for 72 hours and analyzed viral lytic gene expression (Fig 2D and 2E). We found that shRYBP-induced lytic viral gene transcription and protein expression were abrogated in RTA-KO KSHV-infected SLK cells. Since the KSHV lytic cycle depends on RTA and RYBP also represses RTA expression, we propose based on these results that RYBP can inhibit the lytic gene expression cascade by repressing RTA expression.

### RYBP rapidly binds to KSHV genome during chromatinization of viral DNA during *de novo* infection

**Fig 3:**
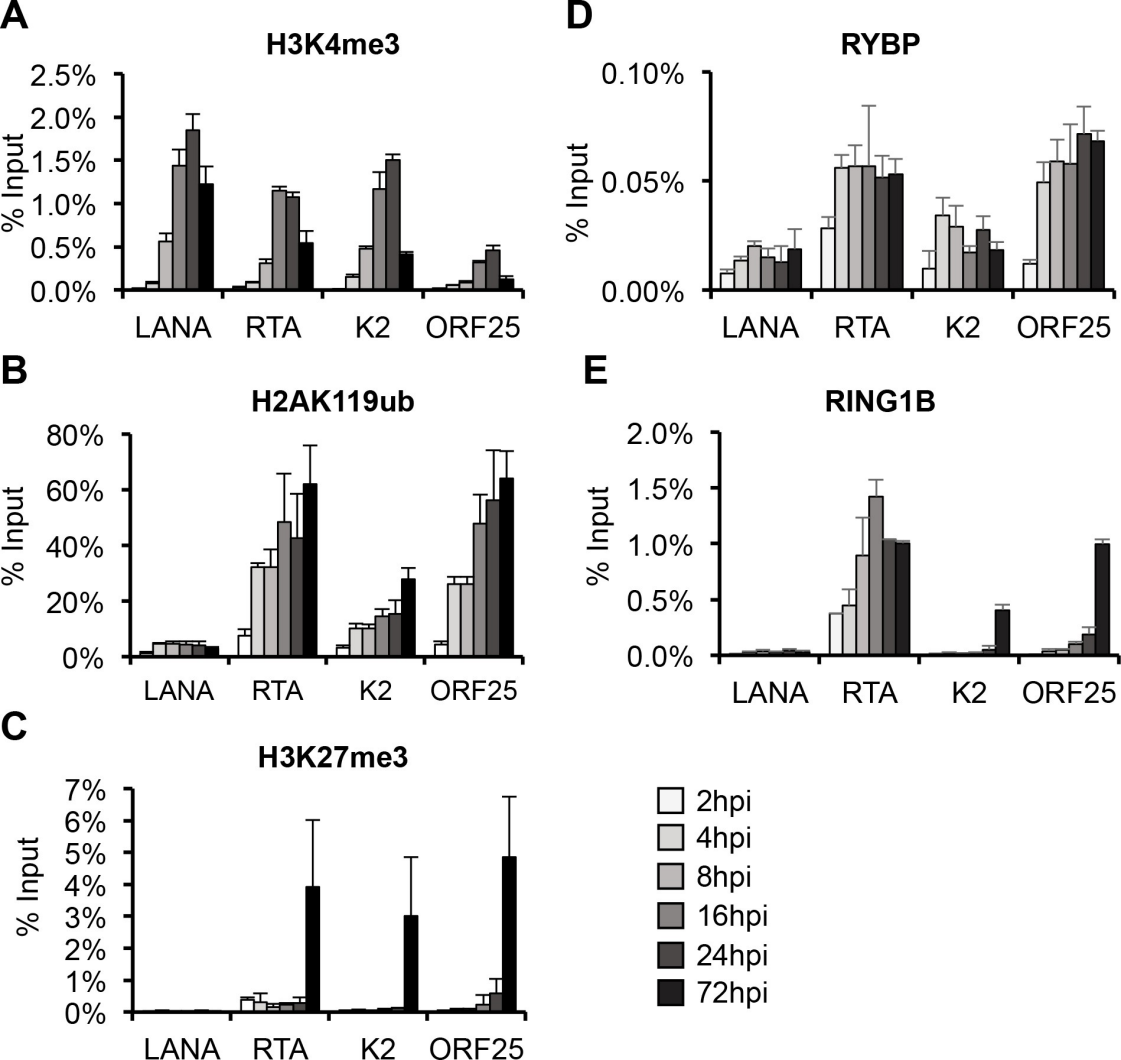
Analysis of the enrichment of histone modifications and the binding of PRC1 factors on KSHV promoters during *de novo* KSHV infection. SLK cells were infected with KSHV, and ChIP-qPCR analysis was performed on LANA, RTA, K2, ORF25 promoters at the indicated time points for **(A)** H3K4me3, **(B)** H2AK119ub, **(C)** H3K27me3, **(D)** RYBP, and **(E)** RING1B.

The fact that RYBP can suppress lytic gene expression in the very early phase of infection indicates that RYBP may rapidly bind to lytic viral promoters during *de novo* infection. To test this hypothesis, time course ChIP experiments were performed in SLK cells during KSHV infection. We analyzed the binding of RYBP, RING1B and the enrichment of histone posttranslational modifications at 2, 4, 8, 16, 24, and 72 hpi at the promoter of LANA, RTA, K2, and ORF25 genes, which are representative of latent, immediate-early, early, and late viral gene classes, respectively. Fig 3 shows that the activating histone mark H3K4me3 and repressive histone marks (H2AK119ub and H3K27me3) exhibited a temporally ordered deposition on the viral promoters, which corroborates data from existing studies [4]. H3K4me3 was enriched on viral promoters by 24 hpi and reduced slightly by 72 hpi (Fig 3A). PRC1-mediated H2AK119ub enrichment on viral lytic gene promoters was observed as early as 4 hpi and then gradually increased over time (Fig 3B). Intriguingly, this data indicates that PRC1-mediated deposition of H2AK119ub can also occur on the KSHV genome in a PRC2-independent manner during *de novo* infection. PRC2-mediated H3K27me3 level was undetectable or very low on the tested viral promoters in the early stage of infection but enriched at 72 hpi (Fig 3C). We found that RYBP binds to KSHV lytic gene promoters as early as 4 hpi, concomitant with the initial enrichment of H3K4me3, while RING1B was detected on RTA promoter at early time points but its binding was delayed at other lytic viral promoters (Fig 3D and 3E). The rapid binding of RYBP was also confirmed at 8 additional KSHV genes during *de novo* infection (S3 Fig). These results not only further confirm the biphasic euchromatin to heterochromatin transition on the KSHV genome following *de novo* infection [4, 36], but also show that PRC1-mediated H2AK119ub can be deposited on viral promoters and RYBP binds to lytic viral genes genome-wide on the KSHV episome as early as 4 hpi. Thus, we found that RYBP recruitment is PRC2 independent and H2AK119ub deposition on viral promoters can also take place prior to the enrichment of PRC2-mediated H3K27me3.

### Neither PRC1’s activity nor PRC1-recruiting factors is required for the binding of RYBP to lytic viral promoters

**Fig 4:**
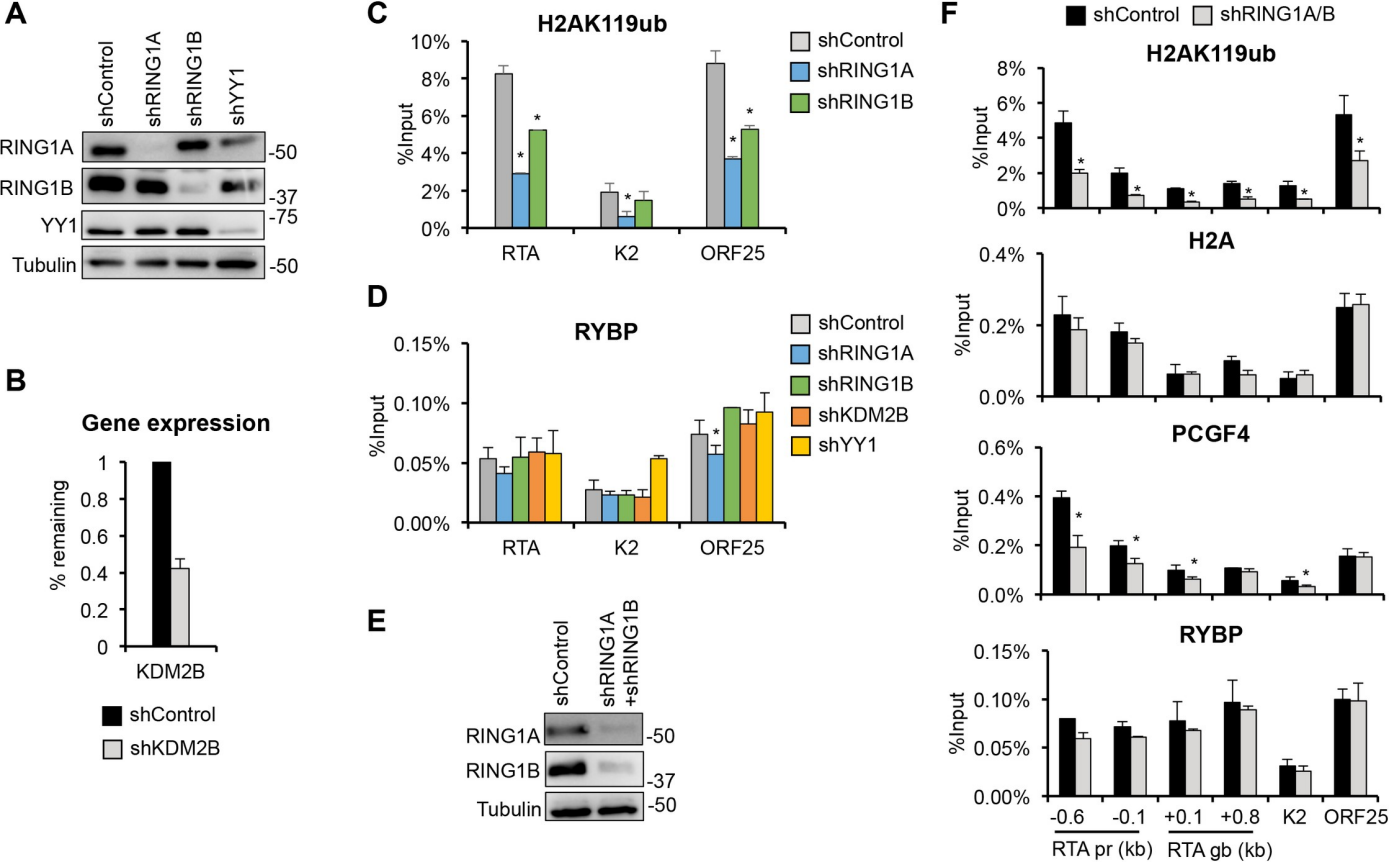
Testing the effect of PRC1 and PRC1-recruiting transcription factors on RYBP binding to KSHV promoters. **(A-D)** SLK cells were transduced with shControl, shRING1A, shRING1B, shKDM2B, and shYY1 lentiviruses for 72 hours followed by KSHV infection for 24 hours. **(A)** Immunoblot analysis to confirm the shRNA knockdown of RING1A, RING1B, and YY1 at the protein level. **(B)** Confirming the shRNA knockdown of KDM2B by RT-qPCR. The percentage of remaining RNA transcript level is shown relative to the shControl. **(C)** ChIP for H2AK119ub. **(D)** ChIP for RYBP. **(E-F)** SLK cells were co-transduced with shRING1A and shRING1B lentiviruses for 72 hours, followed by KSHV infection for 24 hours. **(E)** The shRNA knockdown of RING1A/B was confirmed by immunoblot. **(F)** ChIP for the indicated factors on viral promoters and RTA gene body. The t-test was performed between shControl and shRING1A, shRING1B, shKDM2B, shYY1 and between shControl and shRING1A/B where *p*<0.05 (*) was considered statistically significant.

Since RYBP is a major component of ncPRC1 complexes, we hypothesized that RYBP is recruited to KSHV lytic promoters as part of ncPRC1. Recent studies showed that ncPRC1 can bind to host promoters via DNA-binding factors such as KDM2B or YY1 [29, 30]. Here, we tested whether RYBP is recruited to viral promoters as part of ncPRC1 or if RING1A/B-mediated H2AK119ub is required for RYBP binding (Figs 4 and S4A). SLK cells transduced with lenti-shRING1A, shRING1B, shKDM2B, or shYY1 were infected with KSHV, and ChIP assays were performed at 24 hpi to analyze RYBP binding and PRC1 activity at different viral promoters. Immunoblot and RT-qPCR analysis showed robust shRNA knockdown (Fig 4A and 4B). We found that shRING1A and shRING1B significantly reduced H2AK119ub level on viral lytic promoters (RTA, K2, and ORF25) indicating that PRC1’s enzymatic activity was disrupted (Fig 4C); however, RYBP binding was not affected (Fig 4D). Similarly, shKDM2B and shYY1 also did not alter RYBP binding on the viral promoters (Fig 4D).

Because RING1A and RING1B are functionally redundant in PRC1, we hypothesized that individual shRNA knockdown may not be sufficient to reduce the binding of RYBP on viral promoters. Therefore, we performed a RING1A/B double shRNA knockdown during *de novo* KSHV infection (Figs 4E, 4F and S4A). We found that while H2A occupancy on viral promoters was not affected by shRING1A/B, H2AK119ub enrichment and PCGF4-binding were reduced, indicating that shRING1A/B did diminish PRC1 activity and affect the binding of a PRC1 factor to viral promoters and RTA gene body (Fig 4F). In contrast, shRING1A/B did not reduce RYBP-binding on any of the 12 KSHV genomic sites tested, including both viral promoters and gene bodies (Figs 4F and S4A).

We also used an alternative strategy to examine if RYBP and RING1B can affect each other’s binding on the KSHV genome. SLK cells overexpressing GFP (negative control), 3xFLAG-RYBP, or 3xFLAG-RING1B were infected with KSHV, and RYBP and RING1B binding was analyzed by ChIP assays at lytic gene promoters at 24 hpi (S4B–S4D Fig). We found that the overexpression of RING1B did not recruit more RYBP to the viral promoters or vice versa (S4C and S4D Fig). Overall, our results support the notion that PRC1 activity and PRC1-recruting factors are dispensable for binding of RYBP to KSHV lytic gene promoters.

### Recruitment of RYBP to KSHV lytic viral promoters is independent of its binding to PRC1

**Fig 5:**
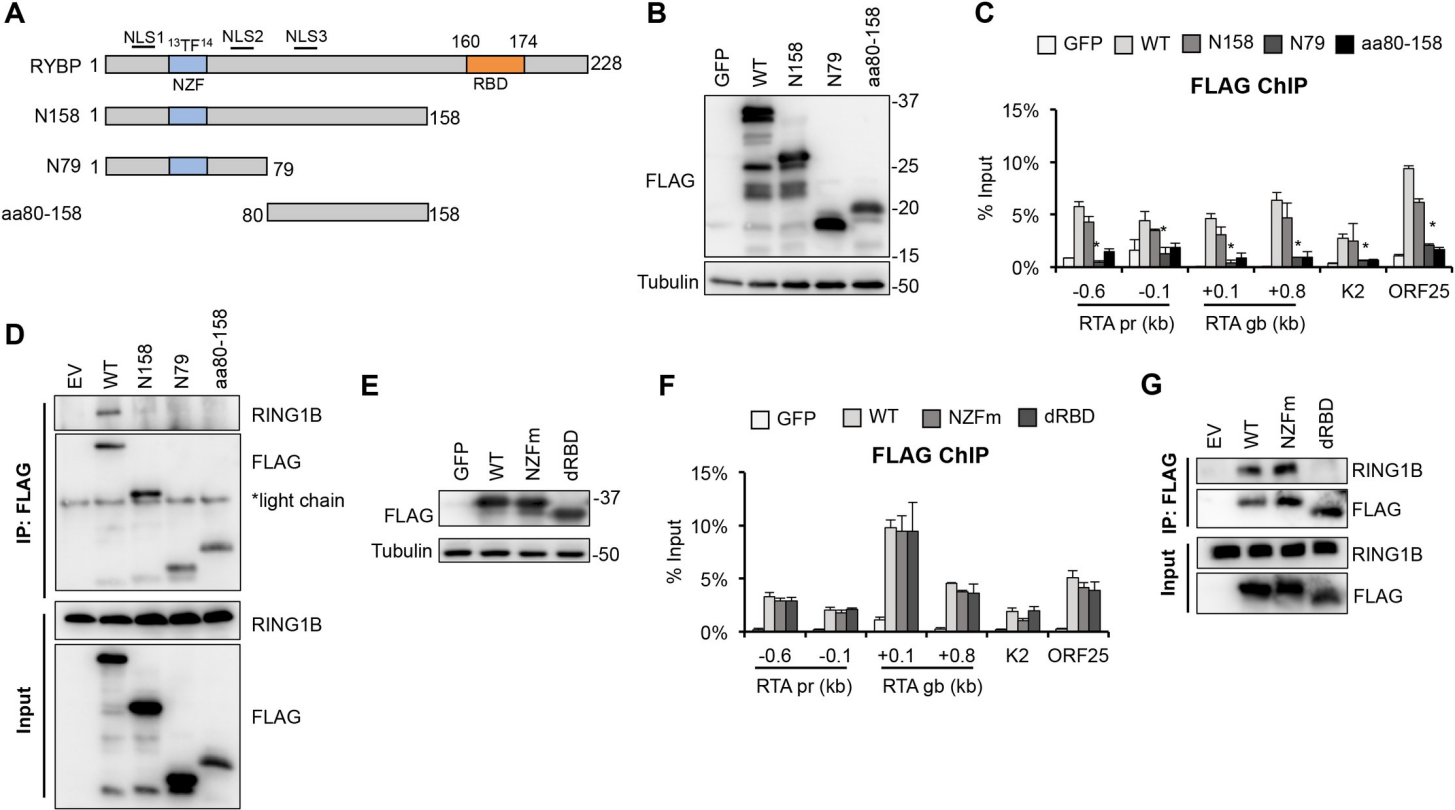
Identification of the functional domain of RYBP that is required for its binding to KSHV promoters. **(A)** Schematic representation of the domain structure of RYBP. NZF, Npl4 Zinc finger motif possessing ubiquitin binding activity; RBD, RING1-binding domain; NLS1-3, nuclear localization signals. Point mutations in NZF mutant (NZFm) are indicated. **(B-C)** SLK cells were transduced with lentiviruses to express GFP (negative control), 3xFLAG-RYBP WT, N158, N79, or aa80-158 RYBP truncation mutants for 3 days, followed by KSHV infection for 24 hours. **(B)** FLAG immunoblot analysis of the expression of WT and truncation mutants of 3xFLAG-RYBP in SLK cells. **(C)** FLAG-ChIP analysis for testing the binding of 3xFLAG-tagged RYBP proteins on viral lytic promoters and RTA gene body. The t-test was performed between 3xFLAG-RYBP and 3xFLAG-RYBP N79 samples, and *p*<0.05 (*) was considered statistically significant. **(D)** FLAG IP using 293T cells expressing 3xFLAG-RYBP WT or truncation mutants. **(E-F)** SLK cells were transduced with lentiviruses expressing GFP, WT, NZFm, or dRBD 3xFLAG-RYBP for 3 days, followed by KSHV infection for 24 hours. **(E)** Immunoblot analysis of the expression of WT and mutants of 3xFLAG-RYBP in SLK cells. **(F)** FLAG ChIP analysis to test the binding of 3xFLAG-tagged RYBP proteins on viral lytic promoters and RTA gene body. Lenti-GFP was used as a negative control in the experiments. **(G)** FLAG IP using 293T cells expressing 3xFLAG-RYBP WT or mutants. EV, empty vector was transfected.

Next, we investigated what part of the RYBP protein is required for its ability to bind to KSHV lytic promoters. RYBP is a 228-amino acid (aa) nuclear protein with three nuclear localization signals (NLS) at the N-terminus and a RING1-binding domain (RBD) at the C-terminus that is required for interaction with PRC1 [31,37]. We constructed three truncation mutants of 3xFLAG-RYBP: N158 (aa1-158), N79 (aa1-79), and aa80-158 (Fig 5A). We confirmed that the expression of WT and RYBP mutants was comparable through immunoblot analysis (Fig 5B).

To analyze the binding of RYBP mutants on KSHV lytic promoters, SLK cells expressing GFP (negative control), 3xFLAG-RYBP (WT) or the RYBP mutants were infected with KSHV, and FLAG ChIP was performed at 24 hpi. The results show that WT RYBP and N158 mutant bound to KSHV lytic gene promoters and RTA gene body, but the N79 and aa80-158 mutants lost the binding ability (Fig 5C**)**. Importantly, we confirmed that the N158 mutant lost its interaction with RING1B in a manner similar to the other RYBP mutants lacking the RBD domain (Fig 5D). This data indicates that RING1B-interaction of RYBP is not required for its binding to KSHV lytic promoters. Since some of the NLSs in N79 and aa80-158 were deleted, we used immunofluorescence assay to examine whether their loss of binding to viral promoters was due to their altered subcellular localization. However, we determined that the RYBP truncation mutants were mostly localized in the nucleus similarly to WT RYBP, although aa80-158 also showed some cytoplasmic staining (S5 Fig). Importantly, we found that the 3xFLAG-RYBP mutant that lacks only the 14-aa RBD still binds to KSHV lytic promoters during *de novo* infection as efficiently as WT RYBP, which confirms that the interaction of RYBP with PRC1 is not required for its binding to KSHV promoters (Fig 5E–5G). Furthermore, our data shows that the NZF domain of RYBP, which mediates RYBP’s interaction with chromatin through binding to H2AK119ub, is also not required for targeting RYBP to lytic viral promoters (Fig 5E–5G) [38,39].

We also tested whether posttranslational modifications of RYBP play a role in its binding to viral promoters during *de novo* infection. RYBP has been shown to undergo phosphorylation at several serine residues whose functions have not yet been characterized [40]. It is known that phosphorylation of Polycomb proteins can modulate their subcellular localization, protein-protein and chromatin interactions, and binding to their target promoters [41]. Therefore, to determine if any of the phosphorylation sites of RYBP affect its binding to viral promoters, we mutated the phosphorylation sites from serine to alanine residues and expressed the mutants as 3xFLAG-tagged proteins in SLK cells during *de novo* KSHV infection. FLAG ChIP analysis at 24 hpi showed that none of the phosphorylation sites of RYBP are required for its binding to KSHV promoters (S6 Fig).

Taken together, these results demonstrate that while PRC1 binding is dispensable, the N-terminus of RYBP is crucial for the binding of RYBP to KSHV lytic promoters during *de novo* infection. This also implies that RYBP may inhibit lytic viral gene expression in a PRC1-independent manner.

### Binding of RYBP to KSHV promoters during *de novo* infection is required for RYBP-mediated repression of lytic genes

Based on the rapid binding of RYBP to lytic viral promoters and its repressive effect on lytic gene expression at 4 hpi, we posited that RYBP binding to viral promoters is required for reducing viral gene expression during *de novo* infection. To test this idea, we overexpressed the 3xFLAG-RYBP mutant N158 during KSHV infection under the assumption that it would compete with the endogenous WT RYBP for binding to KSHV promoters (S7 Fig). It is important to note that the RYBP antibody used for ChIP is specific for the C-terminus of RYBP. Thus, the RYBP ChIP in the presence of N158 overexpression can detect only the binding of endogenous RYBP to viral promoters. Our results show that N158 overexpression reduced endogenous RYBP binding to KSHV lytic gene promoters (RTA and ORF25), while RING1B-binding and H2AK119ub were not affected (S7A–S7C Fig). In addition, RT-qPCR analysis showed that KSHV lytic gene expression was increased by N158 overexpression. These results led us to conclude that full-length RYBP binding is required for RYBP-mediated KSHV gene repression during *de novo* infection.

### RYBP does not affect the chromatinization of KSHV or the binding or function of PRC1, but reduces the level of histone marks linked to transcription elongation

**Fig 6:**
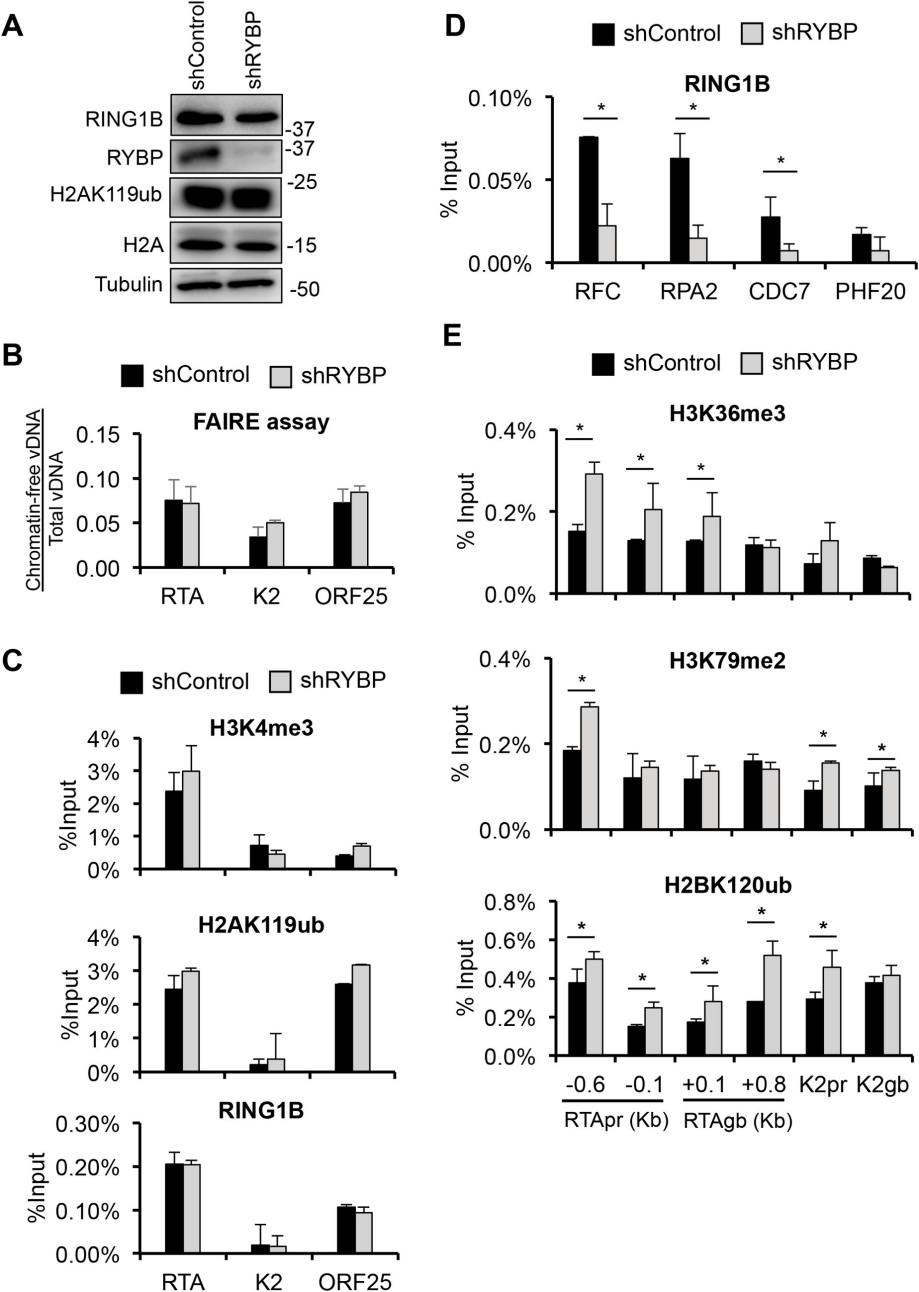
Testing the effect of RYBP depletion on the KSHV epigenome at 24 hpi. shControl- and shRYBP-treated SLK cells were infected with RTA-KO KSHV for 24 hours. **(A)** Immunoblot analysis of host epigenetic factors. **(B)** FAIRE assay to analyze the chromatinization of KSHV promoters during *de novo* KSHV infection. **(C)** ChIP analysis of the enrichment of H3K4me3, H2AK119ub, and RING1B on viral promoters. **(D)** RING1B ChIP on host promoters. **(E)** ChIP analysis of H3K36me3, H3K79me2, and H2BK120ub at viral promoters and on RTA gene body. The t-tests were performed between shControl and shRYBP samples. *p*<0.05 (*) was considered statistically significant.

To determine whether RYBP affects the formation of KSHV epigenome during *de novo* infection, we infected shControl- and shRYBP-treated SLK cells with RTA-KO KSHV and performed a FAIRE assay and ChIPs for different epigenetic factors on viral promoters at 24 hpi (Fig 6). We used RTA-KO KSHV in these experiments to exclude the effects of shRYBP-induced RTA on the chromatinization of KSHV DNA during *de novo* infection. Immunoblot in Fig 6A shows that the global level of RING1B, H2A, and H2AK119ub was not altered by RYBP depletion. The FAIRE assay, which identifies nucleosome-depleted DNA regions and measures chromatin occupancy of a given DNA region [42], showed that shRYBP does not affect the packaging of viral promoter regions into chromatin (Fig 6B). Similarly, shRYBP did not alter the enrichment of H3K4me3 on lytic viral promoters (Fig 6C). It is known that RYBP enhances PRC1’s enzymatic activity and can bind to H2AK119ub thereby recruiting PRC1 to host promoters [32,39]. Strikingly, our ChIP results showed that shRYBP did not change H2AK119ub level or affect RING1B binding on KSHV lytic promoters (Figs 6C and S8), but reduced RING1B-binding at PRC1-regulated cellular promoters (Fig 6D). Thus, we confirmed that RYBP can function as a PRC1 factor on host promoters, but our results imply that RYBP functions differently on KSHV promoters during *de novo* infection. Furthermore, we found that shRYBP increased the level of histone marks that are associated with RNA polymerase II-mediated transcription elongation such as H3K36me3, H3K79me2 and H2BK120ub on several regions of the KSHV genome (Fig 6E).

Taken together, these results show that RYBP does not affect the chromatinization of viral promoters, the binding of RING1B or the deposition of H2AK119ub on viral promoters supporting our notion that the effect of RYBP on KSHV gene regulation during *de novo* infection is PRC1-independent. Although H3K4me3 is considered as a marker of active gene transcription, shRYBP did not affect H3K4me3 level on the tested lytic viral promoters during *de novo* infection. Instead, we found that shRYBP increases the level of transcription elongation-associated histone marks at KSHV lytic genes suggesting that RYBP may inhibit the transcription elongation step of viral gene transcription.

### RYBP downregulates RTA expression by inhibiting transcription elongation at the RTA gene

**Fig 7:**
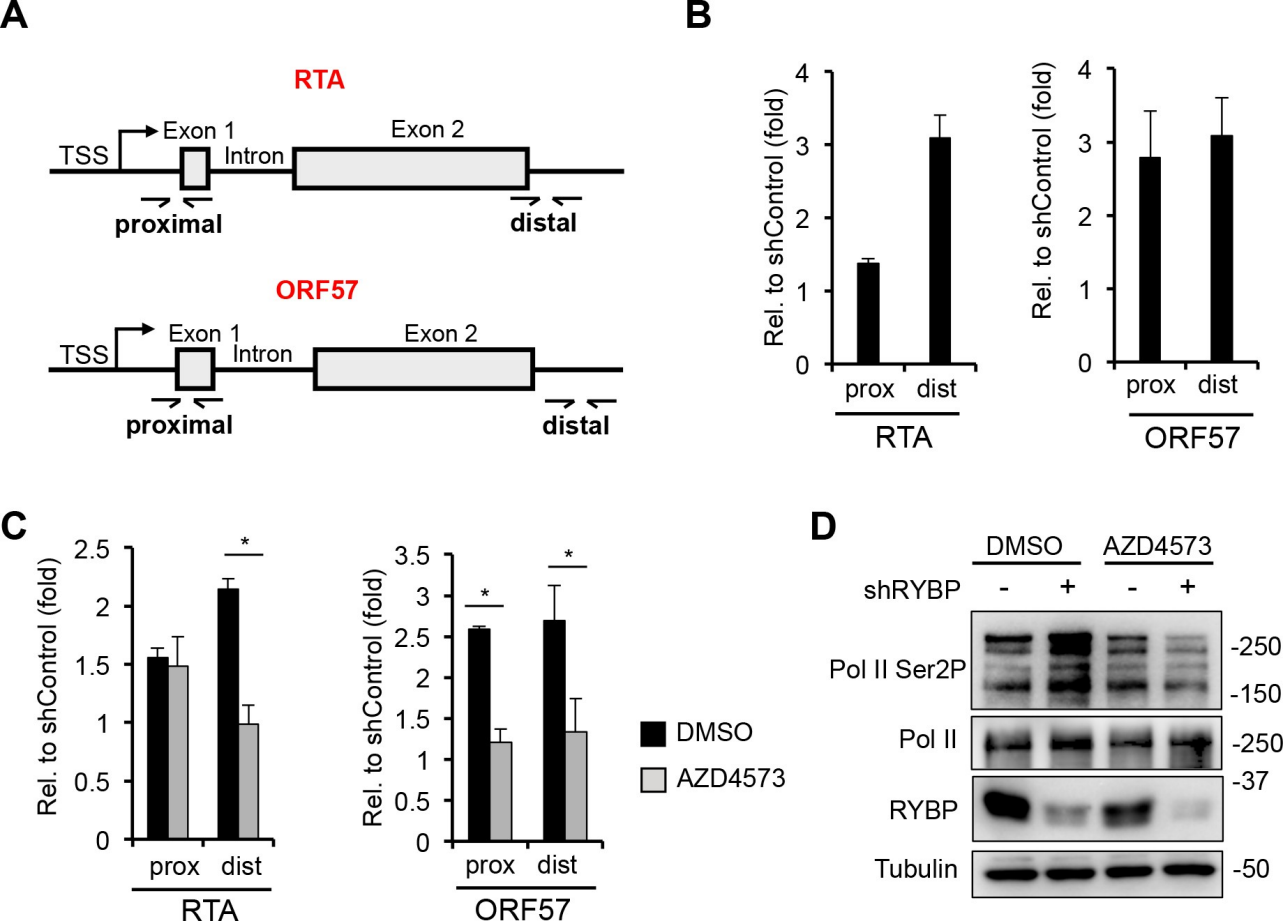
The effect of RYBP on transcription initiation and elongation at viral genes. **(A)** Schematic representation of the structure of RTA and ORF57 genes. The location of RT-qPCR primers targeting the proximal (prox) and distal (dist) regions are indicated. **(B)** SLK cells were transduced with shControl- and shRYBP-expressing lentiviruses for 72 hours, followed by KSHV infection for 8 hours. RT-qPCR analysis of viral mRNA levels at the proximal and distal gene regions of RTA and ORF57 upon RYBP depletion relative to shControl. **(C)** shControl- and shRYBP-treated SLK cells were treated with 30 nM AZD4573 and infected with KSHV for 8 hours. RT-qPCR analysis of viral mRNA levels at the proximal and distal gene regions of RTA and ORF57 upon RYBP depletion relative to shControl samples. The t-test was performed between DMSO-treated and AZD4573-treated shRYBP samples and *p*<0.05 (*) was considered statistically significant. **(D)** Immunoblots analysis of RYBP and Pol II expression as well as Ser2 phosphorylation levels of Pol II.

In the following experiments, we wanted to determine if RYBP can repress the transcription elongation step of KSHV gene expression. To this end, we infected shControl- and shRYBP-treated SLK cells with KSHV and measured the level of viral gene transcription along two lytic viral genes (RTA and ORF57) at their proximal and distal regions relative to the transcription start sites at 8 hpi (Fig 7A). Measuring the mRNA transcript level nearby after the transcription start sites of genes shows the level of transcription initiation, while measuring the gene transcription at the distal gene region indicates the amount of long mRNA transcripts, which reflects the level of transcription elongation at genes. A similar strategy was used to investigate the regulation of transcription elongation of Herpes simplex virus IE genes as well [43]. We found that shRYBP increased the mRNA transcript level at the distal region of RTA substantially (3-fold) but not at the proximal region, suggesting that RYBP inhibits RTA gene transcription mainly at the transcription elongation step (Fig 7B). In contrast, shRYBP increased ORF57 transription similarly at both the proximal and distal regions (Fig 7B), which is consistent with ORF57 being an RTA-inducible gene whose expression depends on RTA expression that is induced by shRYBP. To further confirm the effect of RYBP on transcription elongation, we treated shControl- and shRYBP-SLK cells with AZD4573 during KSHV infection and performed RT-qPCR at 8 hpi to measure gene transcription at the proxmal and distal regions of RTA and ORF57 genes. AZD4573 is a CDK9 inhibitor that blocks CDK9-mediated trancription elongation resulting in reduced RNA transcript level at the disal regions of genes [44]. Fig 7C shows that, upon AZD4573 treatment, shRYBP-induced gene transcription was abrogated at the distal but not the proximal gene region of RTA supporting the idea that RYBP may control the transcription elongation step of RTA gene expression. In contrast, AZD4573 reduced the gene transcription level of ORF57 at both the proximal and distal gene regions of ORF57. Since ORF57 is a RTA-inducable gene, it is possible that the decreased RTA expression by AZD4573 treatment caused the overall defect in its gene transcription (Fig 7C). Interestingly, we also observed that Ser2 phosphorylation of RNA polymerase II, which is mediated by CDK9 and is required for active transcription elongation, was also increased in total cell lysates upon shRYBP treatment. however it was reversed with AZD4573 treatment (Fig 7D). In summary, these data support the notion that RYBP inhibits RTA expression during *de novo* infection by repressing transcription elongation at RTA gene, and that RYBP may have a global role in regulating the transcription elongation form of RNA polymerase II.

## Discussion

The goal of this study was to investigate the role of PRC1 factors in the establishment of viral latency following *de novo* KSHV infection, which is still poorly understood. We tested 7 distinct PRC1 factors during primary KSHV infection and found that each of them contributes to the downregulation of viral lytic genes to differing degrees during infection (Fig 1). However, of these PRC1 factors, we identified RYBP as the most potent repressor of lytic viral genes during *de novo* KSHV infection. We demonstrated that RYBP binds to many different lytic viral promoters on the KSHV genome and inhibits lytic gene expression as early as 4 hpi (Figs 2, 3 and S3). Surprisingly, we found that the binding of RYBP to KSHV genome is PRC1-independent and that RYBP does not affect the binding or function of PRC1 on viral promoters (Figs 4–6 and S4). We also analyzed the effect of RYBP on the KSHV epigenome and found that RYBP does not affect the chromatinization of KSHV genome but instead reduces the enrichment of transcription elongation-associated histone marks on viral lytic genes (Fig 6). Our results suggest that RYBP promotes the establishment of viral latency following *de novo* KSHV infection by inhibiting the expression of the lytic cycle inducer viral gene RTA through blocking its transcription elongation (Fig 7).

Based on our finding that RYBP binds to KSHV lytic promoters concomitantly with H2AK119ub enrichment during the first hours of viral infection prior to PRC2 binding, we assumed that RYBP functions as a ncPRC1 factor on the KSHV genome (Fig 3). This was supported by previous studies that identified RYBP as a major subunit of ncPRC1 complexes that enhances their E3 ubiquitin ligase activity, resulting in increased enrichment of H2AK119ub on host chromatin compared to cPRC1 [26, 39, 45]. Accordingly, the lack of RYBP results in widespread reduction of H2AK119ub at PRC1’s host target sites [39]. Contrary to our expectations, we found that shRNA inhibition of RYBP did not reduce the level of H2K119ub or RING1B binding on KSHV lytic promoters, while it did reduce RING1B binding at PRC1’s host target promoters (Figs 6 and S8). These results indicate that RYBP does not function as a PRC1 factor on the KSHV genome in the first 24 hpi. This conclusion was further supported by our observations that shRING1A/B did not affect RYBP binding on viral promoters. Thus, despite RYBP binding correlates with H2AK119ub enrichment on the KSHV genome and RYBP is known as a major component of ncPRC1, we identified RYBP as a novel PRC1-independent repressor of viral lytic genes during primary KSHV infection. Whether RYBP suppresses lytic gene expression of KSHV during latency in a PRC1-dependent or -independent manner remains to be determined in future studies.

RYBP is a core component of ncPRC1 complexes, and can be recruited to cellular promoters by a number of DNA-binding factors independently of PRC2 [46]. KDM2B is a PRC1 accessory protein that binds to unmethylated CpG islands through its CXXC DNA binding domain and recruits ncPRC1 to cellular promoters [29]. However, our recent study showed that KDM2B is not involved in recruiting PRC1 to KSHV lytic promoters and here we found that it also does not control RYBP binding to viral lytic promoters during *de novo* infection [15]. In addition, we also tested if YY1, another PRC1 recruiting DNA-binding factor at cellular genes, plays any role in RYBP binding on KSHV promoters. We demonstrated that YY1 is also unnecessary for RYBP binding (Fig 4) [30]. There are additional cellular transcription factors, such as MGA, E2F6, and the methylated histone-binding protein L3MBTL2 that can target the non-canonical PRC1.6 complex to specific host promoters [28]; however, we did not further explore these host factors. Based on the PRC1-independent binding of RYBP to KSHV promoters, it is unlikely that any of these PRC1-targeting factors play a role in RYBP recruitment to the KSHV genome.

To determine the region of RYBP that is required for its binding to KSHV promoters, we identified the first 158 amino acids of RYBP, which lacks the C-terminal RING1-binding domain (RBD), that are required for RYBP binding on lytic viral promoters (Fig 5) [31]. In line with this result, we found that the RBD deletion mutant of RYBP was able to bind to KSHV lytic promoters as efficiently as the WT RYBP (Fig 5). It is important to note that the N-terminus of RYBP includes the ubiquitin binding NZF domain whereby RYBP can bind to H2AK119ub and recruit PRC1 to cellular promoters [45,47]. Our data indicates that the NZF mutant can bind to KSHV lytic promoters, while the first 79 residues of RYBP (N79) containing the NZF domain cannot, indicating that the H2AK119ub-binding activity of RYBP is not required for it to interact with the viral promoters (Fig 5). In addition, we also found that the overexpression of RING1B does not increase RYBP level on KSHV promoters and vice versa (S4 Fig). Altogether, our data supports the notion that RYBP binds to KSHV genome in a PRC1-independent manner.

The prevailing view is that RYBP can regulate cellular gene silencing as part of the ncPRC1 complexes, as increasing the enzymatic activity of ncPRC1 complexes promotes the deposition of high amounts of the repressive histone mark H2AK119ub on chromatin independently of PRC2 and H3K27me3 [26,32]. Additionally, PRC1 can also inhibit cellular gene expression through chromatin compaction, disrupting the RNAPII transcription machinery, downregulating RNAPII processivity, or preventing transcription initiation [48–51]. It is important to note that none of these PRC1 functions have been directly linked to RYBP itself. Furthermore, RYBP-mediated gene silencing mechanisms that are independent of PRC1 have not yet been identified. Our ChIP analysis showed that while RYBP does not affect H3K4me3, H2AK119ub, and RING1B binding, it can reduce the level of transcription elongation histone marks H3K36me3, H2BK120ub and H3K79me2 on viral genes during *de novo* KSHV infection to some degree (Fig 6). These histone marks are mediated by distinct histone modifying enzymes associated with RNAPII that can modulate the processivity of RNAPII [52]. The histone methyltransferase SETD2 binds to Ser2 phosphorylated RNAPII and deposits H3K36me3 in the gene bodies to prevent initiation of cryptic transcriptions [53, 54]. H3K79me2 is mediated by DOT1L, which is highly enriched at the 5’ end of genes and cooperates with various transcription elongation complexes [55]. H2BK120ub can be deposited by a number of different E3 ubiquitin ligases and can facilitate the recruitment and function of the histone chaperon FACT to promote transcription elongation [56,57]. Although our current data cannot pinpoint how RYBP inhibits viral transcription by modulating the levels of RNAPII transcription elongation histone marks, we provided further evidence that RYBP may inhibit the transcription elongation step of RTA gene expression. We demonstrated that shRYBP increased the level of long mRNA transcript at RTA gene but only slightly affected the amount of short transcript at the proximal gene region of RTA, which is indicative of the level of transcription initiation (Fig 7). Additionally, the CDK9 inhibitor abolished shRYBP-mediated increased RNA transcript levels at the distal gene region of RTA, supporting the notion that RYBP downregulates RTA gene expression by inhibiting its transcription elongation. Interestingly enough, we also detected a globally increased Ser2 phosphorylation of the RNAPII C-terminal domain upon shRYBP treatment, which was reversed upon addition of a CDK9 inhibitor (Fig 7). Based on these results, we propose that RYBP promotes the establishment of KSHV latency following *de novo* infection through downregulation of the lytic cycle inducer gene RTA expression by blocking its transcription elongation. Future functional analysis will be required to determine what transcription elongation-associated factors RYBP interacts with and how RYBP can regulate RNAPII transcription elongation.

Thus far, six different ncPRC1 complexes have been identified in mammals, all of which include RYBP, while KDM2B has been shown to be a component of the ncPRC1.1 complex [26]. Despite KDM2B and RYBP being shown to regulate the binding and activity of ncPRC1 on cellular promoters, our studies demonstrate that they function independently of PRC1 on the KSHV genome (Fig 6). We found that KDM2B reduces the transcription initiation-associated histone mark H3K4me3 on the RTA promoter during *de novo* KSHV infection while RYBP reduces H3K36me3 and H2BK102ub, both of which are hallmarks of transcription elongation [15]. These results suggest that KDM2B and RYBP inhibit different steps of RTA gene expression during primary KSHV infection, which ensures the efficient downregulation of the lytic cycle inducer gene and reinforcement of the establishment of KSHV latency.

## Supporting information

S1 Table shRNA target sequences. (DOCX)

S2 Table Antibodies used in the study. (DOCX)

S3 Table Sequences of oligos used in the study. (DOCX)

S1 Fig Inhibition of PRC1 activity induces KSHV lytic gene and protein expression during *de novo* infection. SLK cells were pre-treated with different concentrations of the PRC1 inhibitor (PTC209) for 24 hours followed by BAC16-3xFLAG-RTA KSHV infection for 24 hours. **(A)** RT-qPCR analysis of viral gene expression. The relative fold change represents the induction of viral gene expression in PTC-209-treated cells relative to DMSO-treated cells. **(B)** Immunoblots analysis of viral protein ORF45 and RTA expression. RTA is detected by FLAG antibody.
(TIF)

S2 Fig Effect of RYBP depletion on *de novo* KSHV infection in different cell lines and on latently infected BCBL1 cells. **(A-B)** 293T cells were transfected with 10 μM siRNA RYBP for 72 hours, followed by KSHV infection for 24 hours. **(A)** Immunoblot analysis. **(B)** RT-qPCR analysis to determine viral gene expression after RYBP depletion. The relative fold change represents the induction of viral gene expression in siRYBP-treated cells relative to siControl-treated cells. **(C-D)** EA.hy926 cells were transduced with RYBP shRNA lentivirus for 72 hours and then infected with KSHV for 72 hours. **(C)** Immunoblot analysis of RYBP and KSHV ORF45 expression. **(D)** RT-qPCR analysis for viral gene expression after RYBP depletion. The relative fold change represents the induction of viral gene expression in shRYBP-treated cells relative to shControl-treated cells. **(E-F)** BCBL1 cells were transduced with shControl or shRYBP lentivirus for 72 hours. **(E)** Immunoblots analysis of RYBP and viral protein expression. **(F)** RT-qPCR analysis of viral gene expression in shRYBP-treated cells relative to shControl-treated cells.
(TIF)

S3 Fig RYBP binding on the KSHV genome. **(A)** Schematic representation of the linear KSHV genome. RYBP binding was measured by ChIP-qPCR at the indicated genomic sites. **(B)** SLK cells were infected with KSHV and RYBP ChIP analysis was performed at different loci on the KSHV genome at the indicated time points of infection.
(TIF)

S4 Fig Testing if RING1A and RING1B affect RYBP binding to viral promoters and if RYBP and RING1B can affect each other’s binding. **(A)** shRING1A/B-treated SLK cells were infected with KSHV for 24 hours, followed by RYBP ChIP analysis at different loci on the KSHV genome. **(B-C)** SLK cells were transduced with lentiviruses expressing GFP, 3xFLAG-RYBP, or 3xFLAG-RING1B for 3 days, followed by KSHV infection for 24 hours. **(B)** FLAG immunoblot analysis of 3xFLAG-RYBP and 3xFLAG-RING1B overexpression. **(C)** ChIP analysis for RYBP and RING1B binding at RTA, K2, and ORF25 promoters. Lenti-GFP was used as negative control in the experiments. The t-tests were performed between GFP and 3xFLAG-RYBP or 3xFLAG-RING1B. *p*<0.05 (*) was considered statistically significant.
(TIF)

S5 Fig Subcellular localization of WT RYBP and RYBP truncation mutants. HeLa cells transfected with empty vector, 3xFLAG-RYBP WT, and 3xFLAG-RYBP mutants were subjected to immunofluorescence analysis using FLAG antibody (red).
(TIF)

S6 Fig Testing the effect of the phosphorylation sites of RYBP on its binding to KSHV promoters. SLK cells were transduced with GFP, 3xFLAG-RYBP, or 3xFLAG-RYBP phosphorylation mutants for 3 days, followed by KSHV infection for 24 hours. FLAG ChIP analysis was performed to test the binding of 3xFLAG-tagged RYBP proteins on the RTA, K2, and ORF25 promoters. Lenti-GFP was used as a negative control in the experiments.
(TIF)

S7 Fig Effect of the overexpressed N158 RYBP mutant on the binding of endogenous RYBP on KSHV promoters and viral gene expression. SLK cells were transduced with lentiviruses expressing GFP or 3xFLAG-RYBP N158 mutant for 3 days, followed by KSHV infection for 24 hours. ChIP analysis for **(A)** RYBP, **(B)** RING1B, and **(C)** H2AK119ub enrichment on RTA and ORF25 promoters. **(D)** RT-qPCR analysis of viral gene expression. Lenti-GFP was used as a negative control in the experiments. The t-tests were performed between GFP and 3xFLAG-RYBP N158, and *p*<0.05 (*) was considered statistically significant.
(TIF)

S8 Fig Analysis of the effect of RYBP on the binding of RING1B to KSHV promoters. shRYBP-treated SLK cells were infected with KSHV for 24 hours, followed by RING1B ChIP analysis at different loci on the KSHV genome.
(TIF)
