# Supplementary material for: PRC1-independent binding and activity of RYBP on the KSHV genome during de novo infection
Source: PLoS Pathog. 2022 Aug 26;18(8):e1010801. doi: 10.1371/journal.ppat.1010801 (PMC9455864; doi:10.1371/journal.ppat.1010801)
Supplement: S1 Table — (DOCX) [file ppat.1010801.s001.docx]

**Table S1. shRNA target sequences**

| **Target gene** | **Target sequence – forward**  **(5’ to 3’)** |
| --- | --- |
| **PCGF2** | GAGCCACTGAAGGAATACT |
| **CBX4 #1** | AGATGAAGATAGTCAAGAA |
| **CBX4 #2** | AGTACGAGCTCAACAGCAA |
| **CBX7** | GCTGGTTCTGGGAGTTAAAGG |
| **RYBP #1** | GGAAATTAGTCCTAGTGTTAC |
| **RYBP #2** | GCACAGCAGTTGGCAGTAACT |
| **YAF2 #1** | GCAGGTTACTCAGCAGTTTGT |
| **YAF2 #2** | GCTGCCAGTTTACACATTTCT |
| **RING1A** | GCCCTGATCTCTAAGATCTAT |
| **RING1B** | GCTCATCAAGAGAGAGTATTA |
| **KDM2B** | GCATGAAGCAGAGCTGCATCA |
| **YY1** | ATGGTTGTAATAAGAAGTT |
