# Supplementary material for: PRC1-independent binding and activity of RYBP on the KSHV genome during de novo infection
Source: PLoS Pathog. 2022 Aug 26;18(8):e1010801. doi: 10.1371/journal.ppat.1010801 (PMC9455864; doi:10.1371/journal.ppat.1010801)
Supplement: S2 Table — (DOCX) [file ppat.1010801.s002.docx]

| Antibody | Host species | Dilution | Source |
| --- | --- | --- | --- |
| RTA | Rabbit polyclonal | 1/1000 (WB) | Yoshihiro Izumiya, UC Davis |
| ORF6 | Rabbit polyclonal | 1/1000 (WB) | Gary S. Hayward,  Johns Hopkins University |
| ORF45 | Mouse monoclonal | 1/1000 (WB) | Santa Cruz; sc-53883 |
| K8.1 | Mouse monoclonal | 1/1000 (WB) | Santa Cruz; sc-65446 |
| Tubulin | Mouse monoclonal | 1/5000 (WB) | Sigma; T5326 |
| RYBP | Rabbit monoclonal | 1/1000 (WB); 1 µg (ChIP) | Abcam; ab185971 |
| H2A | Rabbit polyclonal | 0.5 µg (ChIP) | Abcam; ab18255 |
| H3K27me3 | Rabbit polyclonal | 0.5 µg (ChIP) | Active Motif; 39155 |
| H3K4me3 | Rabbit polyclonal | 0.5 µg (ChIP) | Active Motif; 39159 |
| H3K36me3 | Rabbit monoclonal | 1 µg (ChIP) | Active Motif; 61022 |
| H3K79me2 | Rabbit polyclonal | 1 µg (ChIP) | Active Motif; 39143 |
| H2AK119ub | Rabbit monoclonal | 1 µg (ChIP) | Cell Signaling; 8240S |
| H2BK120ub | Rabbit monoclonal | 1 µg (ChIP) | Cell Signaling; 5546S |
| RING1B | Rabbit polyclonal | 1/1000 (WB); 1 µg (ChIP) | Abcam; ab3832 |
| RING1A | Rabbit monoclonal | 1/1000 (WB) | Cell Signaling; 13069S |
| YY1 | Mouse monoclonal | 1/1000 (WB) | Santa Cruz; sc7341 |
| PCGF4 | Mouse monoclonal | 1 µg (ChIP) | Santa Cruz; sc390443 |
| RNA Pol-II CTD | Rabbit polyclonal | 1/1000 (WB) | Abcam; ab26721 |
| Pol-II Ser2P | Rabbit polyclonal | 1/1000 (WB) | Abcam; ab5095 |
| FLAG | Mouse monoclonal | 1/1000 (WB); 1 µg (ChIP); 1/400 (IF) | Sigma; F1804 |

**Table S2. Antibodies used in the study**
