# Supplementary material for: PRC1-independent binding and activity of RYBP on the KSHV genome during de novo infection
Source: PLoS Pathog. 2022 Aug 26;18(8):e1010801. doi: 10.1371/journal.ppat.1010801 (PMC9455864; doi:10.1371/journal.ppat.1010801)
Supplement: S3 Table — (DOCX) [file ppat.1010801.s003.docx]

**Table S3. Sequences of oligos used in the study**

| **Gene** | **Forward (5' - 3')** | **Reverse (5' - 3')** | **Application** |
| --- | --- | --- | --- |
| **LANA** | GTTTATAAGTCAGCCGGACCAA | GATATAACTCCGCCCTCCACTA | ChIP-qPCR |
| **RTA (-0.6 kb)** | AAGACACTGACCCACCAAGG | GGTGCCACCAATGTATGACC | ChIP-qPCR |
| **RTA (-0.1 kb)** | AAAGTCAACCTTACTCCGCAAG | GCTGCCTGGACAGTATTCTCAC | ChIP-qPCR |
| **RTA (+0.1 kb)** | GTCTGTTGTGAGAATACTGTCCA | TTACCTTGTCATCTTGCGCCAT | ChIP-qPCR, RT-qPCR |
| **RTA (+0.8 kb)** | TTGCCAAGTTTGTACAACTGCT | ACCTTGCAAAGACCATTCAGAT | ChIP-qPCR |
| **RTA 2^nd^ exon** | CAAGGTGTGCCGTGTAGAGAT | GGTCAAAGCCTTACGCTTCTT | RT-qPCR |
| **RTA 3’ UTR** | ACACTGTACCAGCTGCACCA | GAAGTTAACGCAGGCACAGAC | RT-qPCR |
| **K2pr** | CATACGCAGCCAAGCTATCA | GCTAGCACAGCAAATTGAGA | ChIP-qPCR |
| **K2gb** | TCACTGCGGGTTAATAGGATTT | CATGACGTCCACGTTTATCACT | ChIP-qPCR,  RT-qPCR |
| **ORF25pr** | AGTTGTCGGTGTCTATCTGT | TGCAGAGCGATACGCAGACT | ChIP-qPCR |
| **ORF25gb** | ACAGTTTATGGCACGCATAGTG | GGTTCTCTGAATCTCGTCGTGT | ChIP-qPCR, RT-qPCR |
| **ORF11** | GGCACCCATACAGCTTCTACGA | CGTTTACTACTGCACACTGCA | qPCR |
| **HS1** | TTCCTATTTGCCAAGGCAGT | CTCTTCAGCCATCCCAAGAC | qPCR |
| **ORF45** | CCATACAGCGACCCTGATGA | CCGATTCTCTGACTCAATACT | RT-qPCR |
| **ORF6** | GTTCAAGATACCCTTGTATGACGA | CTTAGAGCCTGTGCTATTCCAGT | RT-qPCR |
| **ORF56** | CACAGATTCCCGTCAATACAAA | GTATCTTCAGTAGGCGGCAGAG | RT-qPCR |
| **ORF36** | ATTGCCAACGACCTGATGCA | ACTCCAGTCCAGCTGCAGCA | ChIP-qPCR, RT-qPCR |
| **RYBP** | CGAAGGGTTTTGGGATTGTA GCGTC | TTGTGCCACCAGCTGAGAATTGATC | RT-qPCR |
| **YAF2** | GGCCTTCAAGTGCATGATGTGCGAT | ACTGCTGAGTAACCTGCTGTGCAAC | RT-qPCR |
| **RING1B** | TACAACGAACACCTCAGGAGGCA | GGCTGTGATGATGCAGTCTGCACA | RT-qPCR |
| **RING1A** | TCTGACTGCATTGTCACAGCCCTA | CAGGCGGATAAGCACTCGGTCTTG | RT-qPCR |
| **PCGF2** | GCTCTGAGTGATGATGAGATTGTC | GCAAGATGCATGACGGTCATGGCT | RT-qPCR |
| **CBX7** | TTTGCACAGAATGAGCTTCG | CAAAAATAAAACCGCGCTCTA | RT-qPCR |
| **CBX4** | CTGGTGAAATGGAGAGGC | GAACGACGGGCAAAGGTAGG | RT-qPCR |
| **ORF8** | CATAGAGCCGAAGGACTGGA | ACCGATAATACCAGCTCTCT | ChIP-qPCR |
| **ORF10** | CTCTTGCGCTATGTGGGACAA | GGCAGTTAGACACCGTTATCT | ChIP-qPCR |
| **K3** | CTAGAGATAGTGAGCCAGGT | CTGGTACGGTCACTTGTTGCA | ChIP-qPCR |
| **K6** | ATGCTGCGTTAGCGTACTGCT | GAACCCGTAGCAGCAGCTAT | ChIP-qPCR |
| **ORF57** | AGGGATATCACCGCTCTCATAAGA | CTGCGGTTTCTCGACGGCAACTCA | ChIP-qPCR |
| **ORF59** | AACCGCAGTTCGTCAGGACCACCA | CCTTAGCCACTTAAGTAGGAATG | ChIP-qPCR |
| **ORF64** | CTTCCTCGAGGGCATCATATAC | TATACGGTGATGGACTTGATGG | ChIP-qPCR, RT-qPCR |
| **18S** | TTCGAACGTCTGCCCTATCAA | GATGTGGTAGCCGTTTCTCAGG | RT-qPCR |
| **KDM2B** | AGTCCGAGACGTCAAACTCCTA | TAGTAACGCACAAACTGGGACA | RT-qPCR |
| **RFCpr** | CTTCGCCTTCTTGCACTTCGC | CAGGATGAAGGCGCTGGCTGG | ChIP-qPCR |
| **RPA2pr** | CCAGAGAAAAGTAGCCAGAGC | CACATCTTGGTCACGATTCTC | ChIP-qPCR |
| **CDC7pr** | CGCATCCGATCGACTCGGTAG | GGAGTACGACTTCTGTGGCTC | ChIP-qPCR |
| **PHD20pr** | AGAGTCCTCTGTCGGTTGGTC | TCTCCTCACGGCCCCACTCCG | ChIP-qPCR |
| **ORF57 5’ end** | AAGGACATGGTACAAGCAATG | CTAGGATGCCCTTCATAATGT | RT-qPCR |
| **ORF57 3’ end** | ATGTAACTCACGTAGCCTTTC | AAGAGACCACGCCTGACTCAT | RT-qPCR |
