# Supplementary figures and images for: PRC1-independent binding and activity of RYBP on the KSHV genome during de novo infection
Source: PLoS Pathog. 2022 Aug 26;18(8):e1010801. doi: 10.1371/journal.ppat.1010801 (PMC9455864; doi:10.1371/journal.ppat.1010801)

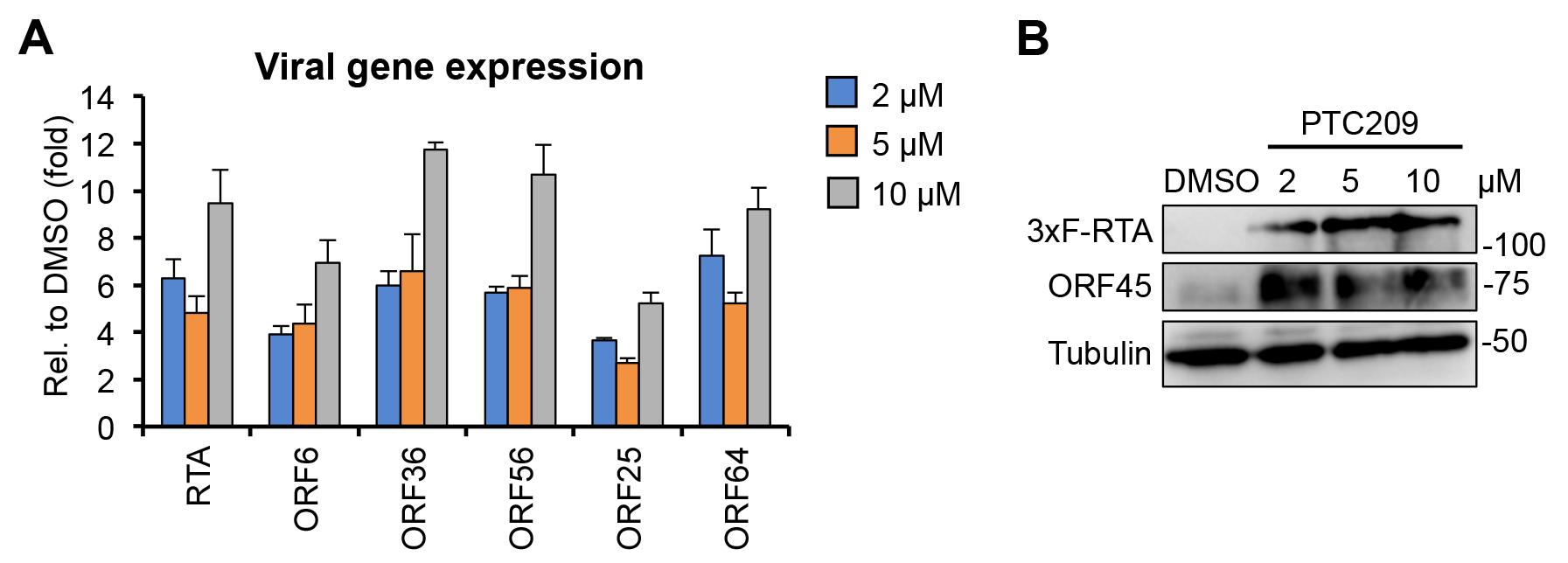

Supplement: S1 Fig — SLK cells were pre-treated with different concentrations of the PRC1 inhibitor (PTC209) for 24 hours followed by BAC16-3xFLAG-RTA KSHV infection for 24 hours. (A) RT-qPCR analysis of viral gene expression. The relative fold change represents the induction of viral gene expression in PTC-209-treated cells relative to DMSO-treated cells. (B) Immunoblots analysis of viral protein ORF45 and RTA expression. RTA is detected by FLAG antibody. (TIF) [file ppat.1010801.s004.tif]

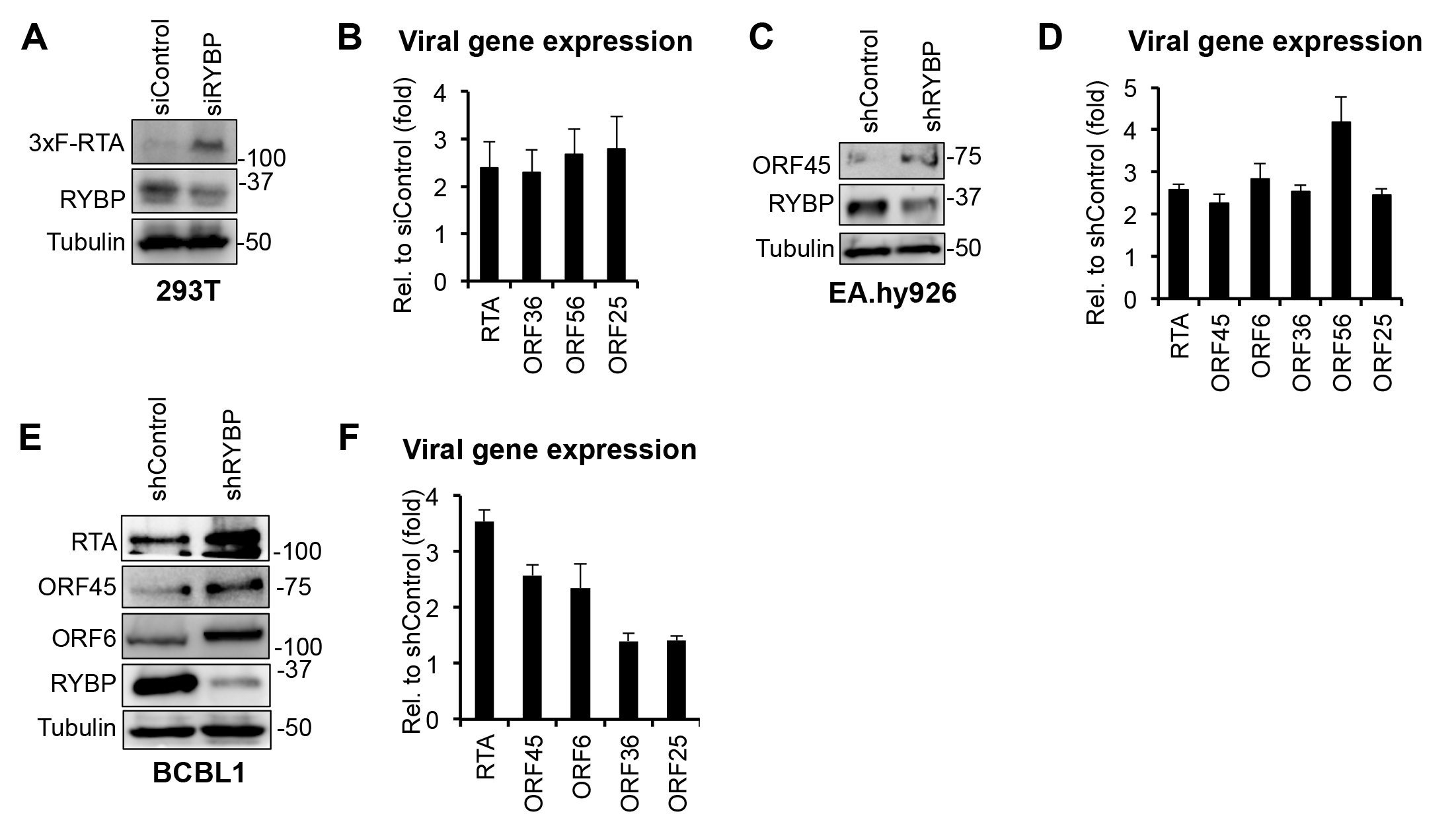

Supplement: S2 Fig — (A-B) 293T cells were transfected with 10 μM siRNA RYBP for 72 hours, followed by KSHV infection for 24 hours. (A) Immunoblot analysis. (B) RT-qPCR analysis to determine viral gene expression after RYBP depletion. The relative fold change represents the induction of viral gene expression in siRYBP-treated cells relative to siControl-treated cells. (C-D) EA.hy926 cells were transduced with RYBP shRNA lentivirus for 72 hours and then infected with KSHV for 72 hours. (C) Immunoblot analysis of RYBP and KSHV ORF45 expression. (D) RT-qPCR analysis for viral gene expression after RYBP depletion. The relative fold change represents the induction of viral gene expression in shRYBP-treated cells relative to shControl-treated cells. (E-F) BCBL1 cells were transduced with shControl or shRYBP lentivirus for 72 hours. (E) Immunoblots analysis of RYBP and viral protein expression. (F) RT-qPCR analysis of viral gene expression in shRYBP-treated cells relative to shControl-treated cells. (TIF) [file ppat.1010801.s005.tif]

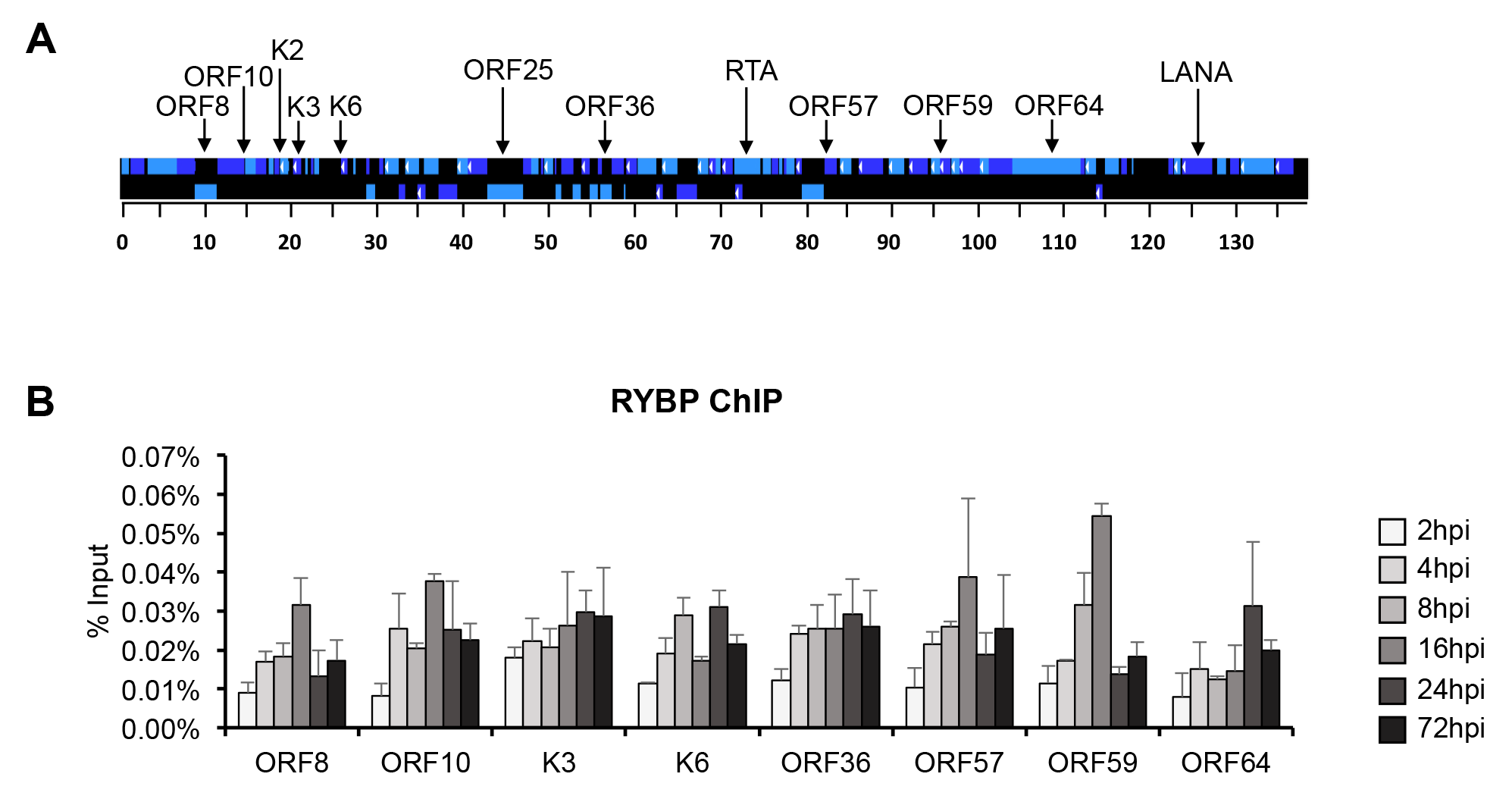

Supplement: S3 Fig — (A) Schematic representation of the linear KSHV genome. RYBP binding was measured by ChIP-qPCR at the indicated genomic sites. (B) SLK cells were infected with KSHV and RYBP ChIP analysis was performed at different loci on the KSHV genome at the indicated time points of infection. (TIF) [file ppat.1010801.s006.tif]

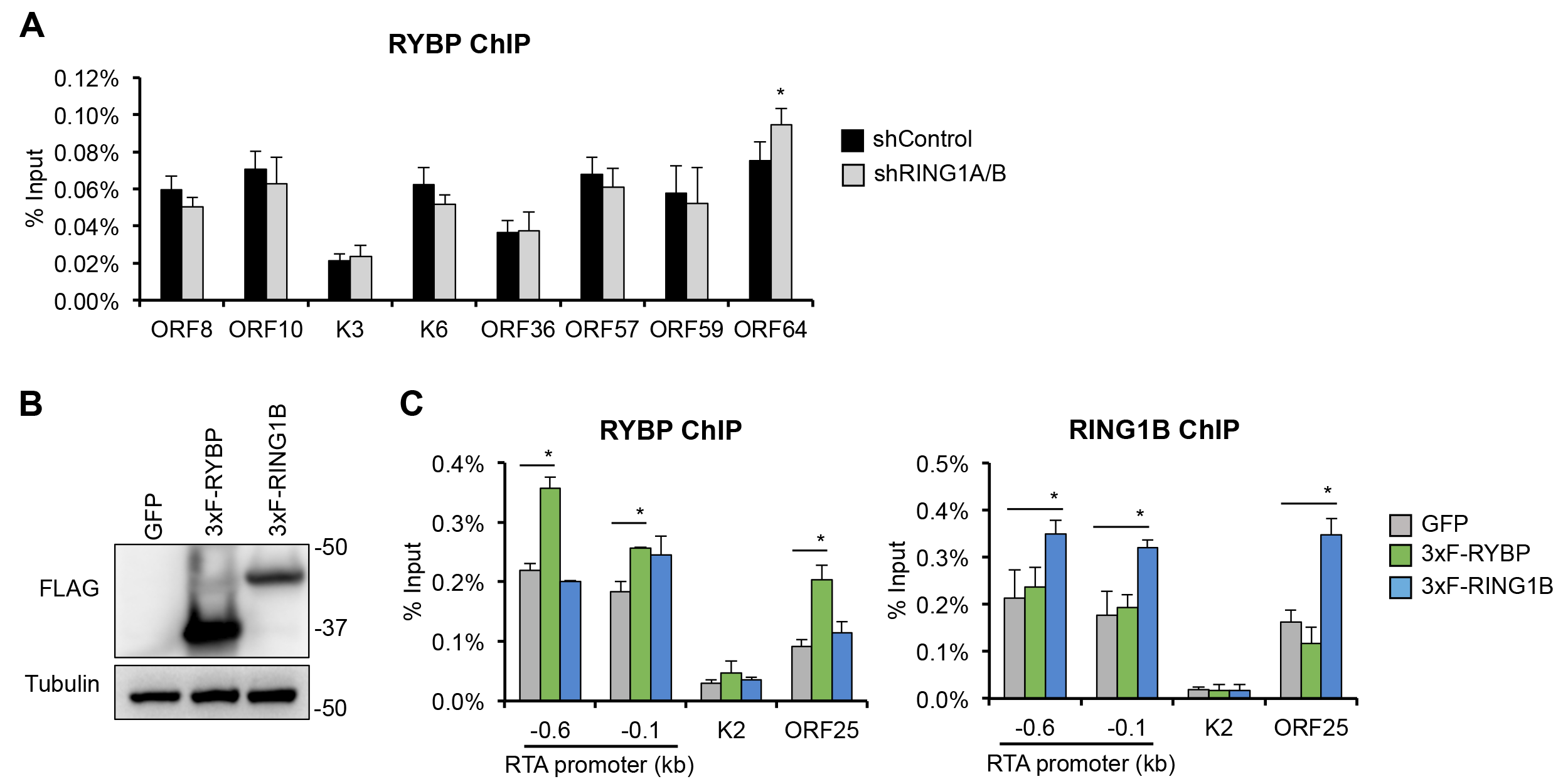

Supplement: S4 Fig — (A) shRING1A/B-treated SLK cells were infected with KSHV for 24 hours, followed by RYBP ChIP analysis at different loci on the KSHV genome. (B-C) SLK cells were transduced with lentiviruses expressing GFP, 3xFLAG-RYBP, or 3xFLAG-RING1B for 3 days, followed by KSHV infection for 24 hours. (B) FLAG immunoblot analysis of 3xFLAG-RYBP and 3xFLAG-RING1B overexpression. (C) ChIP analysis for RYBP and RING1B binding at RTA, K2, and ORF25 promoters. Lenti-GFP was used as negative control in the experiments. The t-tests were performed between GFP and 3xFLAG-RYBP or 3xFLAG-RING1B. p<0.05 (*) was considered statistically significant. (TIF) [file ppat.1010801.s007.tif]

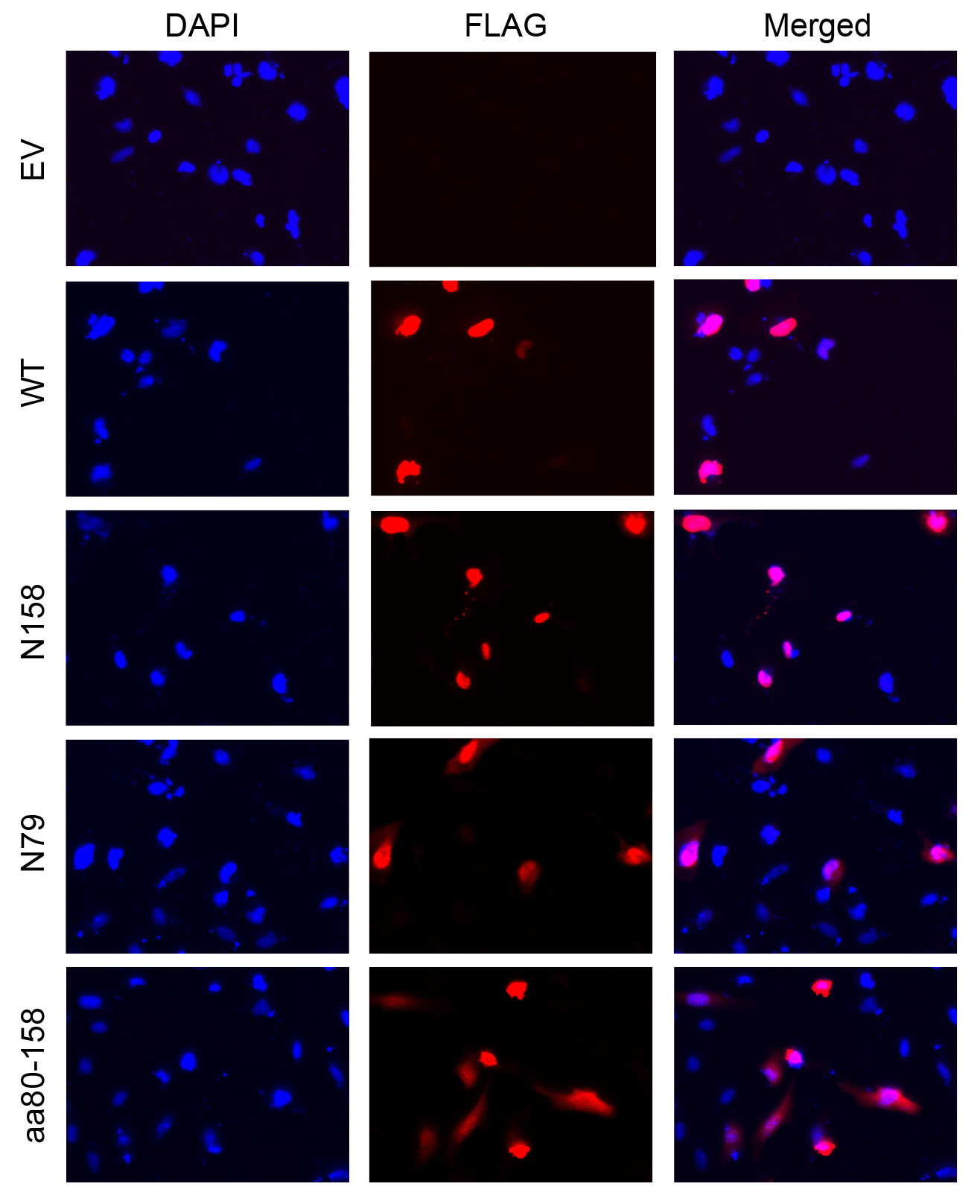

Supplement: S5 Fig — HeLa cells transfected with empty vector, 3xFLAG-RYBP WT, and 3xFLAG-RYBP mutants were subjected to immunofluorescence analysis using FLAG antibody (red). (TIF) [file ppat.1010801.s008.tif]

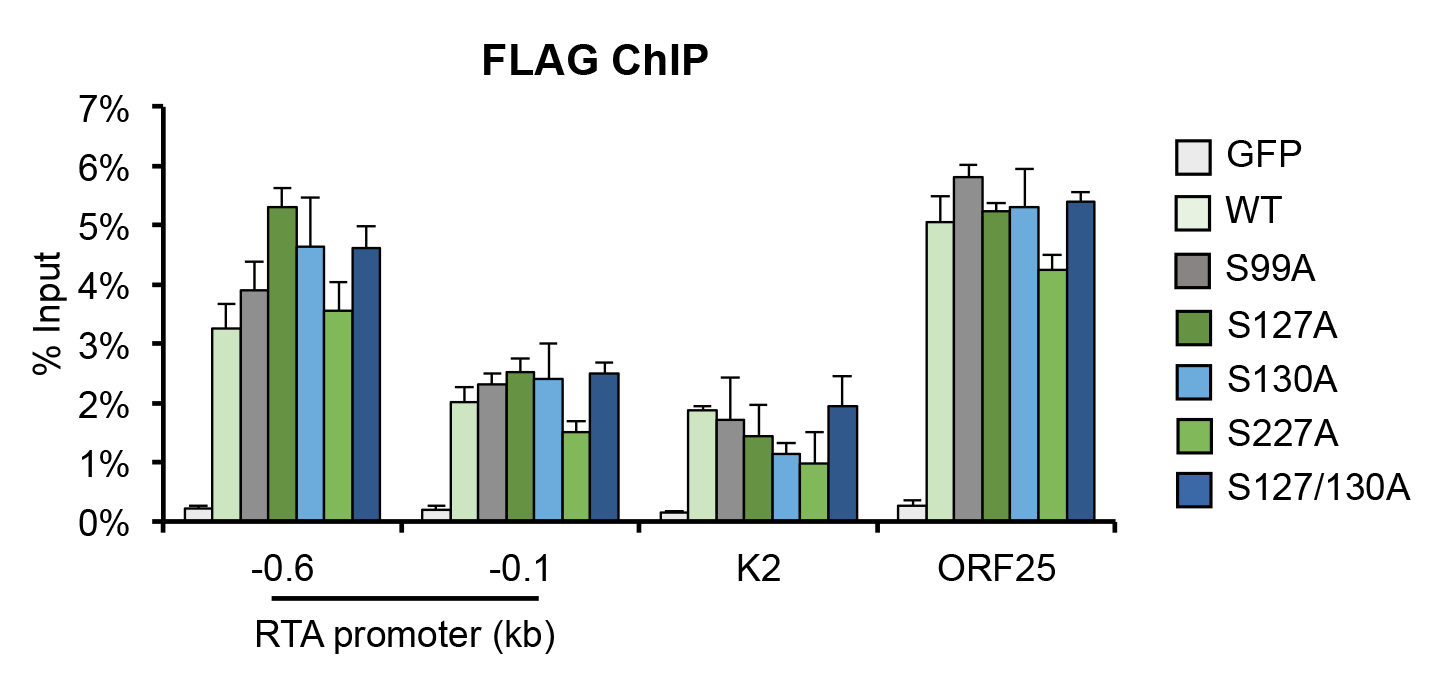

Supplement: S6 Fig — SLK cells were transduced with GFP, 3xFLAG-RYBP, or 3xFLAG-RYBP phosphorylation mutants for 3 days, followed by KSHV infection for 24 hours. FLAG ChIP analysis was performed to test the binding of 3xFLAG-tagged RYBP proteins on the RTA, K2, and ORF25 promoters. Lenti-GFP was used as a negative control in the experiments. (TIF) [file ppat.1010801.s009.tif]

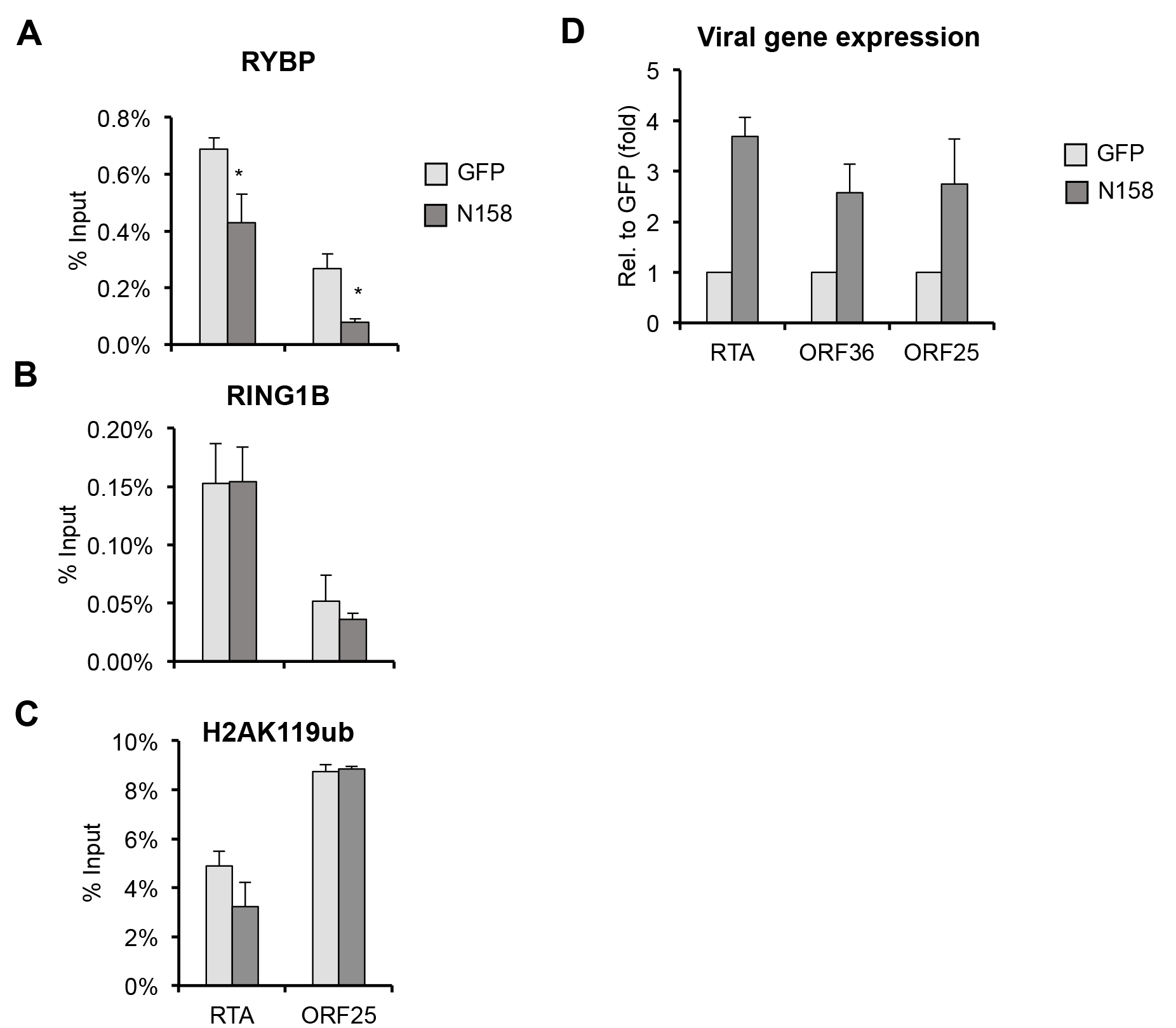

Supplement: S7 Fig — SLK cells were transduced with lentiviruses expressing GFP or 3xFLAG-RYBP N158 mutant for 3 days, followed by KSHV infection for 24 hours. ChIP analysis for (A) RYBP, (B) RING1B, and (C) H2AK119ub enrichment on RTA and ORF25 promoters. (D) RT-qPCR analysis of viral gene expression. Lenti-GFP was used as a negative control in the experiments. The t-tests were performed between GFP and 3xFLAG-RYBP N158, and p<0.05 (*) was considered statistically significant. (TIF) [file ppat.1010801.s010.tif]

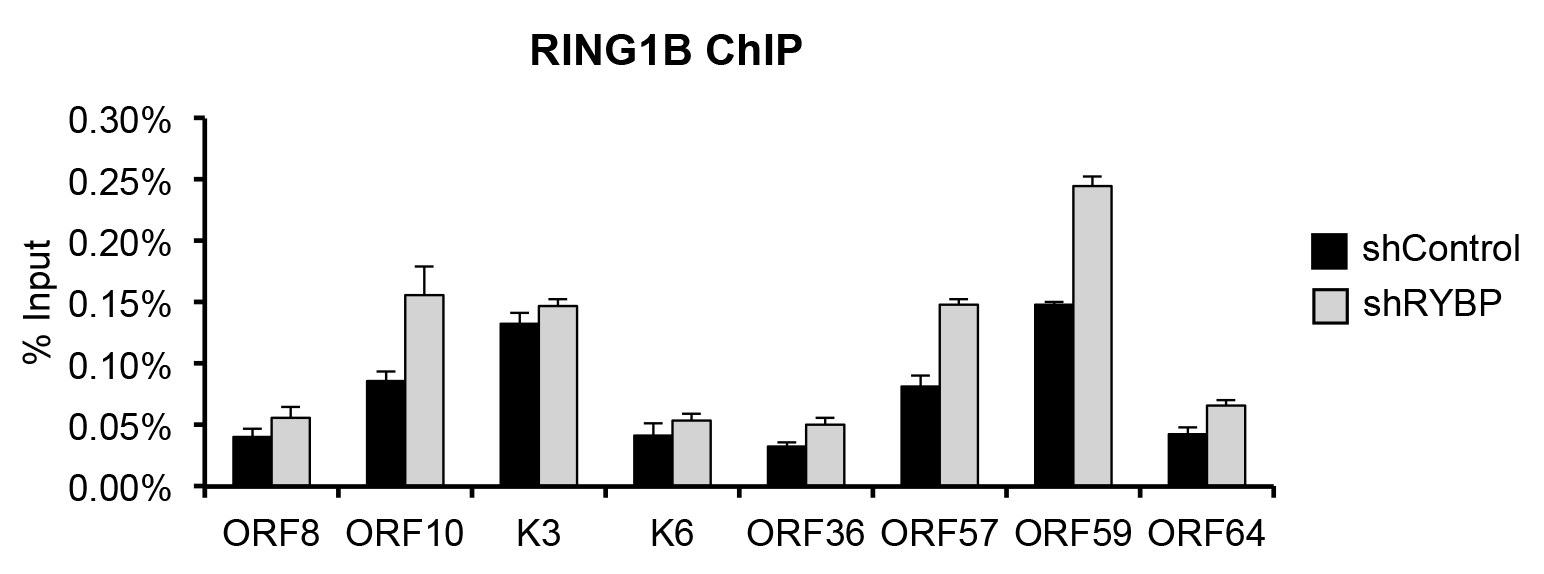

Supplement: S8 Fig — shRYBP-treated SLK cells were infected with KSHV for 24 hours, followed by RING1B ChIP analysis at different loci on the KSHV genome. (TIF) [file ppat.1010801.s011.tif]
